# Stage-specific MSC spheroid fusion enables bottom-up formation of stage-defined hierarchical cartilage graft

**DOI:** 10.1016/j.mtbio.2026.103649

**Published:** 2026-09-04

**Authors:** Yen-Liang Liu, Yun-Chieh Lin, Yi-Chen Tsai, Chun-Che Yen, Chun-Yi Chiang, Pen-Jung Hung, Yu-Ting Huang, Chih-Hung Chang, Feng-Huei Lin, Hwa-Chang Liu

**Affiliations:** a Master Program for Biomedical Engineering, College of Biomedical Engineering, China Medical University, Taichung City, 406040, Taiwan; b Cancer Biology and Precision Therapeutics Center, China Medical University, Taichung City, 406040, Taiwan; c Graduate Institute of Biomedical Sciences, College of Medicine, China Medical University, Taichung City, 406040, Taiwan; d Department of Orthopaedic Surgery, National Taiwan University Hospital, Taipei City, 100225, Taiwan; e Department of Biomedical Engineering, College of Biomedical Engineering, China Medical University, Taichung City, 406040, Taiwan; f Kartigen Biomedical Inc., Taipei City, 100047, Taiwan; g Ph.D. Program for Aging, College of Medicine, China Medical University, Taichung City, 406040, Taiwan; h Department of Orthopaedic Surgery, Far Eastern Memorial Hospital, New Taipei City, 220216, Taiwan; i Graduate School of Biotechnology and Bioengineering, Yuan Ze University, Taoyuan City, 320315, Taiwan; j Department of Biomedical Engineering, College of Engineering, National Taiwan University, Taipei City, 106319, Taiwan; k Institute of Biomedical Engineering and Nanomedicine, National Health Research Institutes, Miaoli County, 350401, Taiwan; l Department of Orthopaedic Surgery, Taiwan Adventist Hospital, Taipei City, 105404, Taiwan

**Keywords:** Cartilage defect, Umbilical cord mesenchymal stem cell, Bone marrow mesenchymal stem cell, Chondrocyte-precursor, Spheroid

## Abstract

Engineering articular cartilage with hierarchical architecture and effective interfacial integration remains a major challenge. Although bottom-up spheroid assembly offers a promising biofabrication strategy, how chondrogenic maturation influences spheroid fusion and boundary remodeling remains incompletely defined. Here, we investigated differentiation stage as a programmable determinant of the phenotype and assembly behavior of mesenchymal stem cell (MSC)-derived chondrogenic spheroids. Exploratory RNA-seq pathway mapping, complemented by biologically replicated RT-qPCR, indicated stage- and cell-source-associated differences in cartilage matrix and maturation-related programs in bone marrow-derived MSCs (BM-MSCs) and umbilical cord-derived MSCs (UC-MSCs). Early-stage spheroids exhibited greater fusion capacity than mature spheroids, whereas incorporation of undifferentiated MSCs promoted the integration of mature chondrogenic spheroids and reduced persistent inter-spheroid boundaries. Sequential assembly of Day-3, Day-7, and Day-14 spheroids generated hierarchical constructs with stage-associated regional differences in cartilage extracellular matrix deposition. Incorporating an undifferentiated MSC layer at the bone-facing interface further supported structural continuity with a porous bone substitute in vitro. In an exploratory porcine pilot study involving two animals, implantation of MSC-derived spheroid grafts into cartilage defects was technically feasible. At 6 months, the repair tissue exhibited variable proteoglycan staining and heterogeneous collagen type II immunoreactivity, while selected sections showed tissue apposition at the graft–host interface. The limited animal-level sample size precluded conclusions regarding repair efficacy, cartilage quality, or superiority over autologous cartilage implantation. Collectively, these findings identify differentiation stage as an intrinsic bioassembly variable and establish a stage-programmed strategy for constructing hierarchical cartilage grafts. Both BM-MSCs and UC-MSCs supported stage-defined assembly in vitro, warranting further evaluation of UC-MSCs as a potential allogeneic cell source in adequately powered studies.

## Highlights

• This study presents a method for fabricating stage-defined hierarchical cartilage grafts based on chondrogenic spheroids.
• The chondrogenic spheroid mosaic combines MSCs and chondrogenic spheroids to promote spheroid integration.
• UC-MSCs and BM-MSCs were compared for chondrogenesis, spheroid integration, and cartilage repair in a porcine model.

## Graphical abstract

**Figure:**
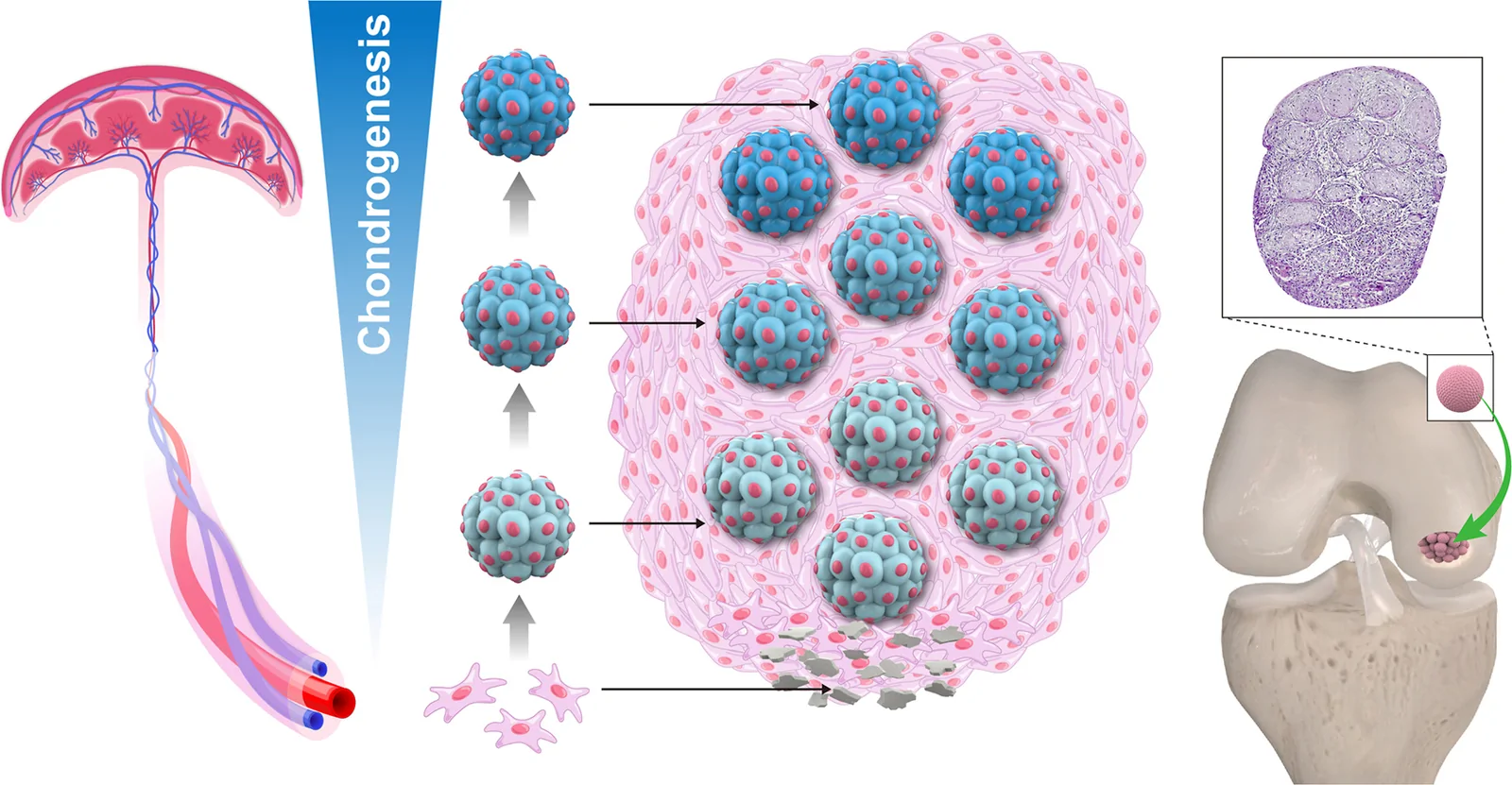

## 1 Introduction

Articular cartilage possesses a highly specialized hierarchical organization composed of superficial, transitional, radial, and calcified zones that collectively enable low-friction articulation and load distribution [1,2]. Loss of this structural anisotropy following trauma or osteoarthritis leads to progressive degeneration and impaired joint biomechanics [3,4]. Although surgical approaches such as microfracture [5,6], mosaicplasty [7,8], and autologous chondrocyte implantation (ACI) [9,10] and matrix-induced autologous chondrocyte implantation (MACI) [11] can improve symptoms, they frequently generate fibrocartilage or structurally homogeneous repair tissue that fails to recapitulate native zonal architecture and long-term mechanical functionality.

To address these limitations, biomaterial-based and cell-based strategies have been developed to engineer osteochondral constructs [12,13]. Multilayered hydrogels [14], porous scaffolds [15], ceramics [16], and bioprinted constructs [17] have been designed to mimic zonal stratification. However, top-down scaffold fabrication often relies on bulk biomaterials to impose morphology, which can limit cell distribution, impair mass transport, and compromise integration with host cartilage [18,19]. In addition, large-volume constructs are prone to hypoxia-induced necrosis and insufficient interfacial bonding [20,21]. These challenges highlight the need for assembly strategies that leverage intrinsic cellular behaviors rather than relying solely on exogenous scaffold architecture.

Bottom-up tissue engineering has emerged as an alternative paradigm in which living building blocks, such as cell spheroids, microtissues, or organoids, are assembled into complex tissues [22,23]. This approach better preserves cell–cell interactions and enhances extracellular matrix (ECM) deposition. Previous studies have demonstrated important advances in zonal cartilage and osteochondral engineering. Mesenchymal condensation has been used to generate large, stratified, and mechanically functional cartilage [24,25], while spheroid and micropellet assembly has enabled the formation of cartilaginous and biphasic osteochondral tissues [26]. Moreover, developmentally programmed organoids representing early, mature, and prehypertrophic phenotypes have been spatially assembled to produce zone-specific osteochondral-like tissues in vivo [27]. Collectively, these studies establish the feasibility of generating zonally defined tissues using modular cellular building blocks.

Recent studies have further defined the biological and biophysical parameters governing spheroid-based cartilage assembly. Sargenti et al. identified spheroid mass density as a potential indicator of chondrogenic maturation and demonstrated its sensitivity to the culture environment [28]. Burdis et al. showed that temporal treatment with chondroitinase ABC promoted matrix remodeling and fusion between adjacent cartilage microtissues, producing a more continuous tissue with a zonally organized collagen network [29]. Kopinski-Grünwald et al. developed scaffolded spheroids containing porous microscaffolds that preserved the bioassembly capacity of mature chondrogenic spheroids and reduced the shrinkage associated with conventional scaffold-free units [30]. More recently, Spagnuolo et al. demonstrated that both microtissue maturation and packing density influenced fusion and subsequent graft development. Less mature Day-2 microtissues fused more rapidly and exhibited greater N-cadherin expression than mature microtissues [31]. Collectively, these findings establish maturation state as both a biological determinant of cartilage phenotype and an engineering determinant of bioassembly.

Thus, the remaining challenge is not whether zonally defined cartilage can be assembled, but how to preserve interfacial integration as the cellular building blocks undergo chondrogenic maturation. Most previous approaches have focused on the spatial specification of tissue phenotypes or have introduced enzymatic or scaffold-based interventions, whereas the use of developmental-stage heterogeneity as an intrinsic cellular strategy for promoting fusion remains insufficiently explored.

This relationship exposes a central design constraint. Progressive chondrogenic maturation promotes cartilage-associated ECM deposition but simultaneously reduces cellular plasticity and matrix-remodeling capacity, potentially leaving persistent boundaries between assembled units [[29], [30], [31], [32]]. Interface formation is particularly challenging in cartilage because its low cellularity and dense, proteoglycan-rich ECM restrict cell migration and matrix interpenetration across graft–graft and graft–host boundaries [26]. Enzymatic matrix remodeling and microscaffold-assisted spheroids can improve fusion [29,30], but these approaches require additional biochemical treatments or material components. It remains unclear whether cellular building blocks at different maturation stages can instead be combined to biologically reconcile matrix formation with interfacial integration.

Developmental morphogenesis provides a rationale for such a strategy. During early chondrogenesis, mesenchymal cells undergo condensation through cell adhesion, cell–cell signaling, and dynamic cellular rearrangement [33]. These early condensations remain plastic and remodeling-competent before progressive differentiation and ECM deposition stabilize the developing tissue [24,30]. Spheroids at different stages may therefore provide complementary assembly functions. Mature chondrogenic spheroids can contribute an established cartilage-associated matrix, whereas undifferentiated mesenchymal stem cells (MSCs) and early-stage spheroids may retain the adhesion, migration, and remodeling capacity required for interfacial integration. Developmental-stage heterogeneity could thus serve as an intrinsic biofabrication parameter rather than simply a source of biological variability.

Cell source represents an additional determinant of this process. Bone marrow–derived MSCs (BM-MSCs) have been widely studied and validated in clinical trials [34,35], yet autologous approaches are limited by donor morbidity, cost, and patient-to-patient variability. Umbilical cord–derived MSCs (UC-MSCs) exhibit high proliferative capacity [36], reduced baseline immunogenicity [37,38], and immunomodulatory properties [39,40], making them promising candidates for allogeneic, off-the-shelf applications. Although both BM-MSCs and UC-MSCs can undergo chondrogenic differentiation [36,41], comparative molecular profiling during staged maturation and their implications for tissue assembly behavior remain incompletely defined.

Here, we investigated the differentiation stage as a programmable determinant of both cartilage-associated phenotype and bioassembly capacity. We first used exploratory transcriptomic pathway mapping and biologically replicated RT-qPCR to characterize BM-MSC- and UC-MSC-derived spheroids during chondrogenic induction. We then quantified differentiation-stage-dependent fusion dynamics and examined whether immature MSC populations could facilitate the integration of mature chondrogenic spheroids. These findings informed the sequential assembly of stage-defined multilayered constructs containing Day-3, Day-7, and Day-14 spheroids, together with an undifferentiated MSC layer at the bone-facing interface. Finally, an exploratory orthotopic porcine cartilage-defect study was conducted to assess surgical feasibility, graft retention, safety, and descriptive tissue responses. By treating developmental stage as an intrinsic biofabrication variable, this study addresses the trade-off between chondrogenic maturation and interfacial remodeling and presents a stage-programmed strategy for engineering hierarchical cartilage grafts.

## 2 Results

### 2.1 Differentiation-stage maturation establishes distinct extracellular matrix programs in MSC-derived spheroids

**Fig. 1:**
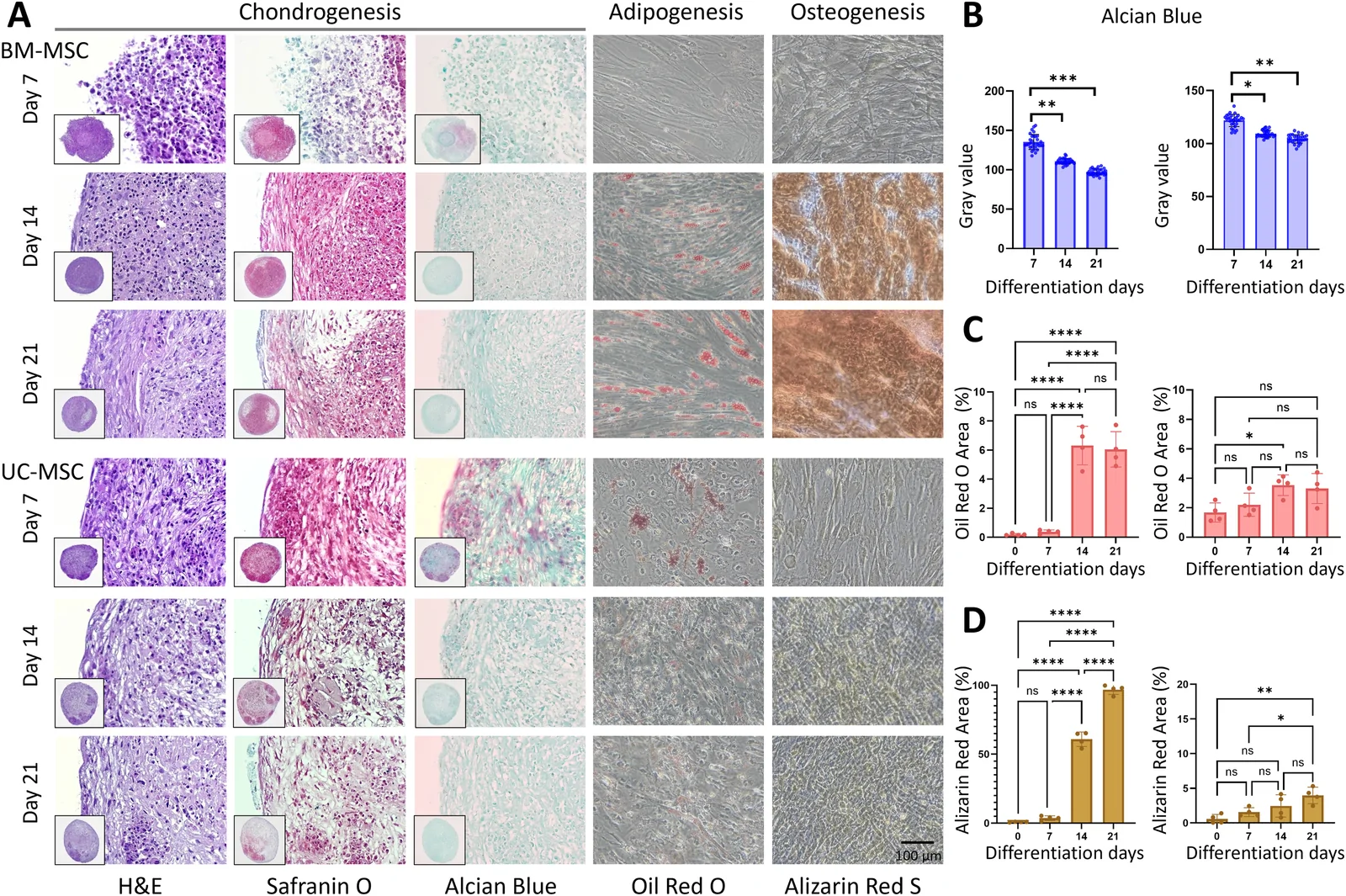
**Trilineage differentiation of BM-MSCs and UC-MSCs**. (A) BM-MSCs and UC-MSCs were cultured under chondrogenic, adipogenic, or osteogenic differentiation conditions for 7, 14, and 21 days. Chondrogenic differentiation was evaluated using Safranin O and Alcian Blue staining, adipogenic differentiation using Oil Red O staining, and osteogenic differentiation using Alizarin Red S staining. (B–D) Quantification of Alcian Blue, Oil Red O, and Alizarin Red S staining, respectively. Alcian Blue staining intensity was quantified using ImageJ and is presented as mean ± SD. Thirty ROIs were analyzed from each of three independent spheroids per group, yielding 90 ROI-level subsampling measurements per group. Statistical comparisons were performed using nested one-way ANOVA with Tukey correction. The biological sample size was n = 3 spheroids per group; the 90 ROIs were not treated as independent biological replicates. Oil Red O- and Alizarin Red S-positive areas were quantified from four fields of view per group. Two-tailed *t*-test; *p < 0.05, **p < 0.01, ***p < 0.001, and ****p < 0.0001.

To determine whether maturation stage influences the molecular phenotype of MSC-derived chondrogenic spheroids, we first characterized BM-MSCs and UC-MSCs. Both cell types displayed typical MSC morphology and expressed CD90 while lacking CD34, consistent with MSC identity (Fig. S1). Trilineage differentiation confirmed adipogenic, osteogenic, and chondrogenic potential in both sources (Fig. 1).

We next evaluated stage-dependent chondrogenic maturation. Spheroids cultured for 7, 14, and 21 days in chondrogenic medium exhibited progressive accumulation of glycosaminoglycans (GAGs) as assessed by Alcian Blue staining (Fig. 1A). Quantitative image analysis demonstrated significantly higher GAGs deposition in Day-21 spheroids compared to earlier stages (Fig. 1B).

### 2.2 Differentiation-stage maturation establishes distinct extracellular matrix programs in MSC-derived spheroids

**Fig. 2:**
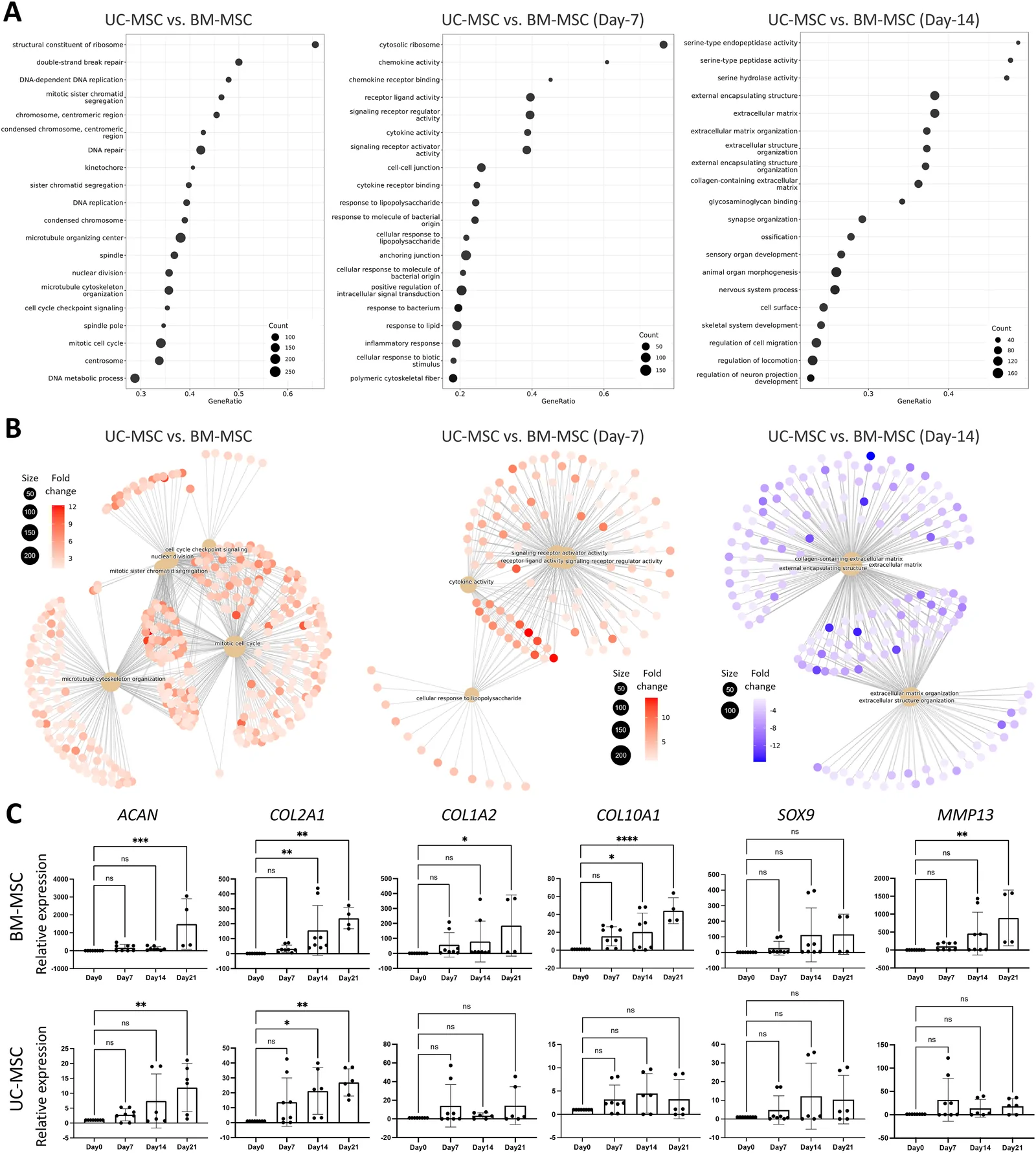
**Exploratory transcriptomic profiling and replicated RT-qPCR analysis of chondrogenic MSC differentiation**. (A) Exploratory Gene Ontology (GO) biological-process enrichment plots derived from descriptive bulk RNA-seq contrasts between UC-MSC- and BM-MSC-derived spheroids. Results are shown for the overall UC-MSC versus BM-MSC comparison and separately at Days 7 and 14 of chondrogenic induction. Dot size indicates gene count. One RNA sample was sequenced for each cell-source and time-point condition (n = 1 per condition); therefore, these enrichment results are exploratory and do not represent biologically replicated differential-expression inference. (B) Category–gene network (cnetplot) of enriched GO terms showing core gene–process relationships underlying chondrogenic differentiation. (C) Independent RT-qPCR analysis of ACAN, COL2A1, COL1A2, COL10A1, SOX9, and MMP13 expression in BM-MSC- and UC-MSC-derived spheroids after 0, 7, 14, and 21 days of chondrogenic induction. Expression was normalized to GAPDH and presented relative to the corresponding undifferentiated Day-0 MSC group. Data are presented as mean ± SD from at least four independent biological replicates per group (n ≥ 4). Statistical comparisons with the corresponding Day-0 group were performed using two-tailed t-tests. ns, not significant; *p < 0.05; **p < 0.01; ***p < 0.001; ****p < 0.0001.

To explore transcriptional differences during chondrogenic differentiation, bulk RNA sequencing was performed on BM-MSC- and UC-MSC-derived spheroids after 7 and 14 days of induction (Fig. 2). One RNA sample was analyzed for each cell-source and time-point condition (n = 1 per condition). Accordingly, the RNA-seq data were used for descriptive pathway mapping and candidate-gene prioritization rather than biologically replicated differential-expression inference.

Gene Ontology (GO) enrichment mapping of the descriptive UC-MSC versus BM-MSC contrasts revealed comparison- and time-dependent patterns (Fig. 2A). The overall contrast was characterized predominantly by terms related to DNA replication, chromosome organization, and cell-cycle regulation. At Day 7, the identified terms included cytokine and chemokine signaling, cell-junction organization, inflammatory responses, and cytoskeletal regulation. By Day 14, the descriptive contrast was dominated by processes associated with extracellular matrix organization, glycosaminoglycan binding, skeletal system development, and ossification. These observations suggest a temporal transition from proliferative and signaling-associated programs toward matrix- and tissue-maturation-associated programs; however, they should be interpreted as exploratory because biological RNA-seq replicates were unavailable.

Category–gene network analysis illustrated the relationships between selected GO terms and their associated genes (Fig. 2B). The descriptive fold-change patterns indicated that individual genes contributed to multiple related pathways, consistent with coordinated regulation of extracellular matrix production and remodeling. Candidate matrix- and remodeling-associated genes identified through this exploratory analysis included COL3A1, MMP2, MMP14, BGN, CEMIP, SERPINE1, SPARC, and THBS1 (Fig. S2). These genes were used to contextualize and prioritize subsequent analyses and were not classified as statistically validated differentially expressed genes.

Independent, biologically replicated RT-qPCR was subsequently used to examine the temporal expression of selected chondrogenic, matrix-related, and hypertrophy-associated genes (Fig. 2C). Relative to undifferentiated Day-0 BM-MSCs, BM-MSC-derived spheroids showed significant induction of *ACAN*, *COL2A1*, *COL1A2*, *COL10A1*, and *MMP13* at one or more time points, whereas the change in *SOX9* expression was not statistically significant. In UC-MSC-derived spheroids, *ACAN* and *COL2A1* were significantly induced at later time points, while changes in *COL1A2*, *COL10A1*, *SOX9*, and *MMP13* did not reach statistical significance. Thus, the replicated RT-qPCR results demonstrate cell-source-specific temporal patterns of chondrogenic and maturation-associated gene expression, while the RNA-seq analysis provides exploratory pathway-level context for these findings.

### 2.3 Differentiation stage governs spheroid fusion dynamics

**Fig. 3:**
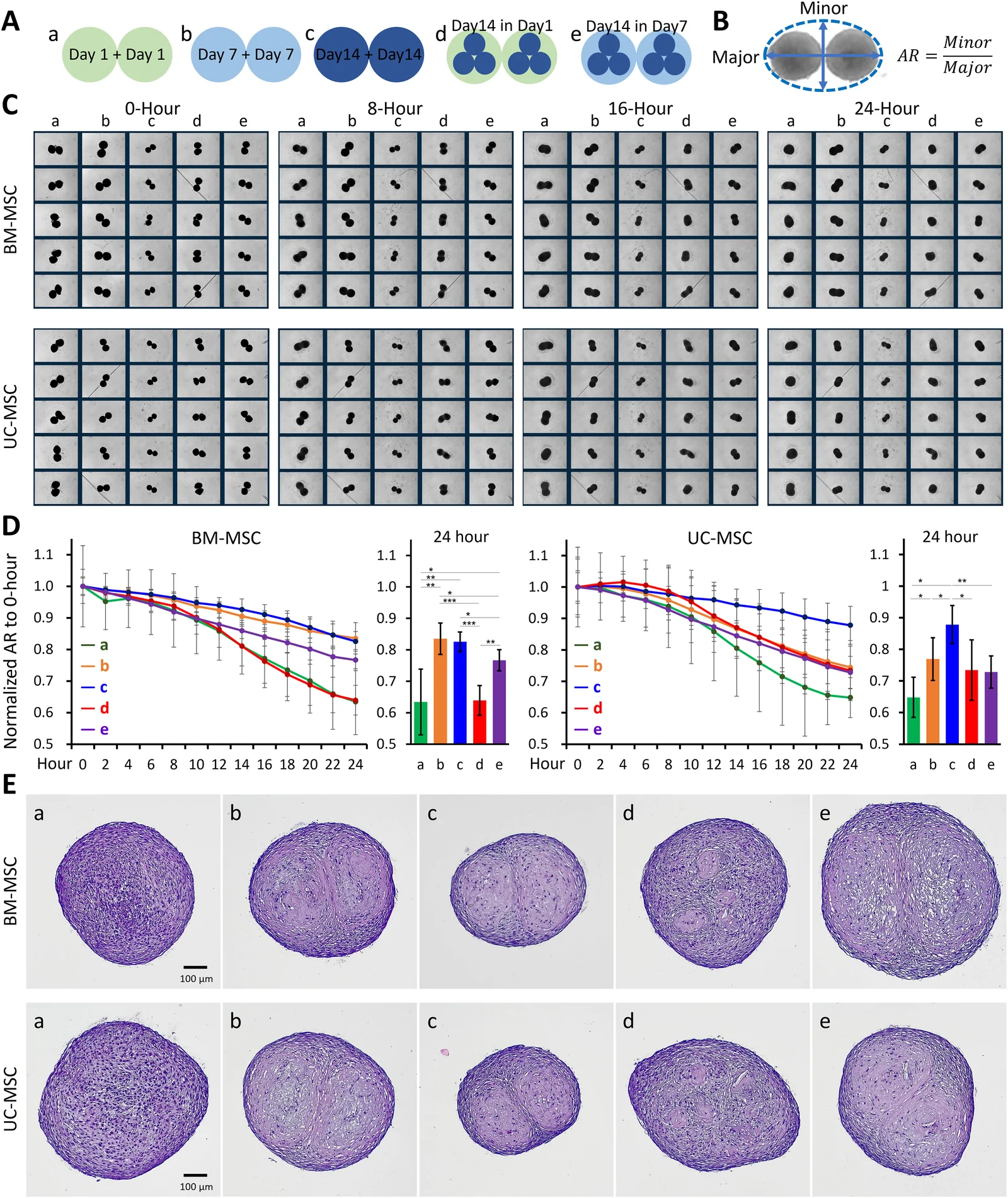
**Chondrogenic spheroid fusion**. (A) Experimental designs of pairs of chondrogenic spheroids for the spheroid fusion assay. Chondrogenic spheroids were independently induced for 1, 7, and 14 days and then paired for the fusion assay. The composite spheroids were prepared by wrapping chondrogenic spheroids with MSCs and then continuing chondrogenesis for specific days. (B) The measurement of the aspect ratio of fusing spheroids. (C) Time-lapse imaging of spheroid fusion (n = 5 biological replicates). (D) Changes of aspect ratios (ARs) during spheroid fusion. (E) H&E staining of fused spheroids.

Given the maturation-dependent differences in matrix remodeling signatures, we next tested whether spheroid fusion behavior is stage-dependent. Pairs of spheroids induced for 1, 7, or 14 days were placed in contact and monitored by time-lapse imaging (Fig. 3A–C).

Early-stage Day-1 spheroids exhibited rapid boundary remodeling and shape relaxation, reflected by a more rapid change in normalized aspect ratio toward a rounded configuration. In contrast, Day-7 and Day-14 spheroids showed delayed shape relaxation and retained more apparent inter-spheroid boundaries during the observation period (Fig. 3D). H&E staining further demonstrated residual interfaces between the more mature paired spheroids (Fig. 3E). Consistent with these observations, qualitative Ridge Detection overlays showed minimal residual separation in Day-1 spheroids and increasingly discernible central interfaces in Day-7 and Day-14 spheroids derived from both BM-MSCs and UC-MSCs (Fig. S6). Ridge Detection was used only to visualize line-like histological boundaries and was not treated as a quantitative fusion endpoint.

To determine whether immature cells could modulate the fusion behavior of mature spheroids, Day-14 spheroids were wrapped with undifferentiated MSCs or early-stage spheroids to generate composite constructs. Notably, composite spheroids restored fusion capacity, with accelerated aspect ratio reduction and diminished histological boundaries relative to mature spheroids alone (Fig. 3D and E).

Together, these findings indicate that differentiation-stage heterogeneity influences spheroid fusion dynamics and that immature MSC populations can facilitate the integration of more mature chondrogenic spheroids. This stage-dependent behavior is consistent with the exploratory transcriptomic patterns associated with extracellular-matrix deposition and remodeling.

### 2.4 Spatial assembly of stage-defined spheroids generates hierarchical cartilage-like constructs

**Fig. 4:**
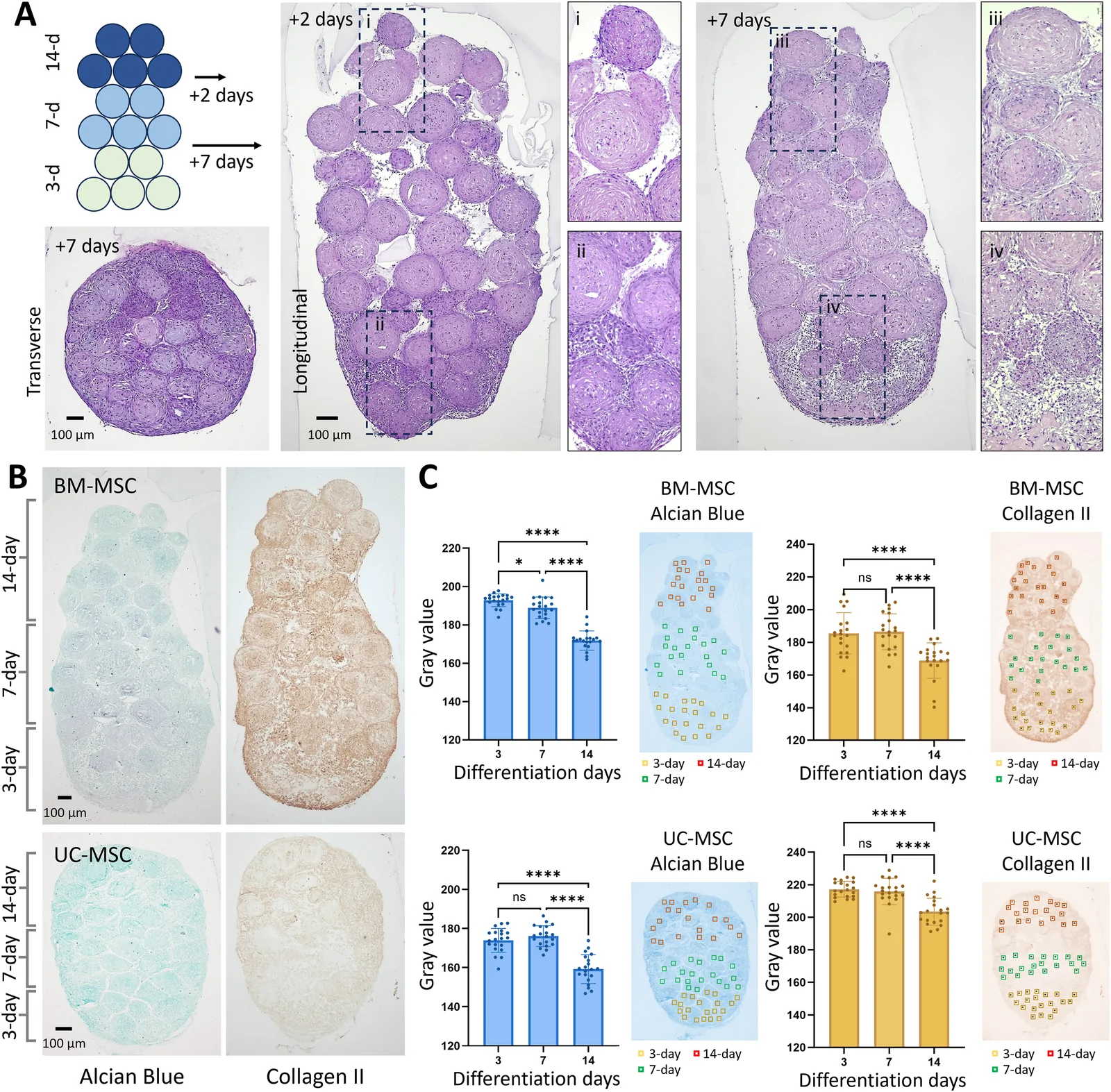
**In vitro assembly of stage-defined chondrogenic spheroids into a three-layer cartilage-like construct**. (A) Schematic illustration of the sequential assembly of Day-3, Day-7, and Day-14 chondrogenic spheroids. Transverse and longitudinal H&E sections show the architecture of the assembled construct. (B) Histological characterization of BM-MSC- and UC-MSC-derived constructs using Alcian Blue staining and collagen type II immunohistochemistry. (C) Quantification of Alcian Blue and collagen type II staining across the predefined regions. Data are presented as mean ± SD (n = 20 measurements per slide; two-tailed *t*-test; *p < 0.05 and ****p < 0.0001).

Having established that spheroids at different stages of chondrogenic induction possessed distinct matrix phenotypes and fusion capacities, we next investigated whether these pre-established properties could be spatially organized within a hierarchical construct. Day-3, Day-7, and Day-14 spheroids were generated separately and then sequentially assembled in a predefined order (Fig. 4A). Day-3, Day-7, and Day-14 spheroids were selected to represent early, intermediate, and relatively mature stages of chondrogenic development, respectively. Day-3 spheroids had undergone sufficient condensation for reproducible handling and assembly while retaining an immature and remodeling-competent phenotype. Day-7 spheroids represented an intermediate stage of chondrogenic commitment and ECM deposition, whereas Day-14 spheroids exhibited a more developed cartilage-associated matrix but reduced fusion capacity. These stages were therefore used to balance the structural stability required for fabrication with the differentiation-stage-dependent plasticity required for inter-spheroid integration. Following assembly, all layers were maintained under the same chondrogenic culture conditions for an additional 2 or 7 days.

Histological analysis demonstrated spatially heterogeneous cartilage ECM deposition across the assembled constructs (Fig. 4B). The regional differences corresponded to the predefined positions of spheroids that had undergone different periods of chondrogenic induction before assembly. Alcian Blue staining and collagen type II immunohistochemistry confirmed cartilage-like matrix deposition throughout the constructs, while quantitative analysis identified differences among the predefined regions (Fig. 4C). These results indicate that spheroids with pre-established stage-associated phenotypes can be spatially assembled and can retain regional differences during subsequent culture. More information about the histology and staining of BM-MSC and UC-MSC chondrogenic spheroid mosaics is shown in Figs. S3 and S4, and independent repeats are shown in Fig. S5.

The upper region containing Day-14 spheroids exhibited greater GAG accumulation than the regions containing less-mature spheroids. Because native articular cartilage normally exhibits lower GAG content in the superficial zone and progressively greater GAG content toward the deep zone, the matrix distribution in the present construct should not be interpreted as a complete reproduction of native articular-cartilage zonation. Instead, the construct represents a multilayer cartilage-like tissue containing spatially organized, stage-associated matrix differences.

Previous studies have similarly shown that spatial assembly of phenotypically distinct cartilaginous organoids or microtissues can preserve predefined regional functions and generate patterned cartilage or osteochondral tissues [27,42]. However, the final matrix distribution in the present constructs may not be determined exclusively by the initial spheroid differentiation stages. Oxygen, nutrient, and soluble-factor gradients may develop during culture of the assembled tissue, and the porous bone substitute may influence the microenvironment at the basal interface. Thus, the observed organization most likely reflects the combined effects of the pre-established spheroid phenotypes, their spatial arrangement, and the local culture microenvironment.

**Fig. 5:**
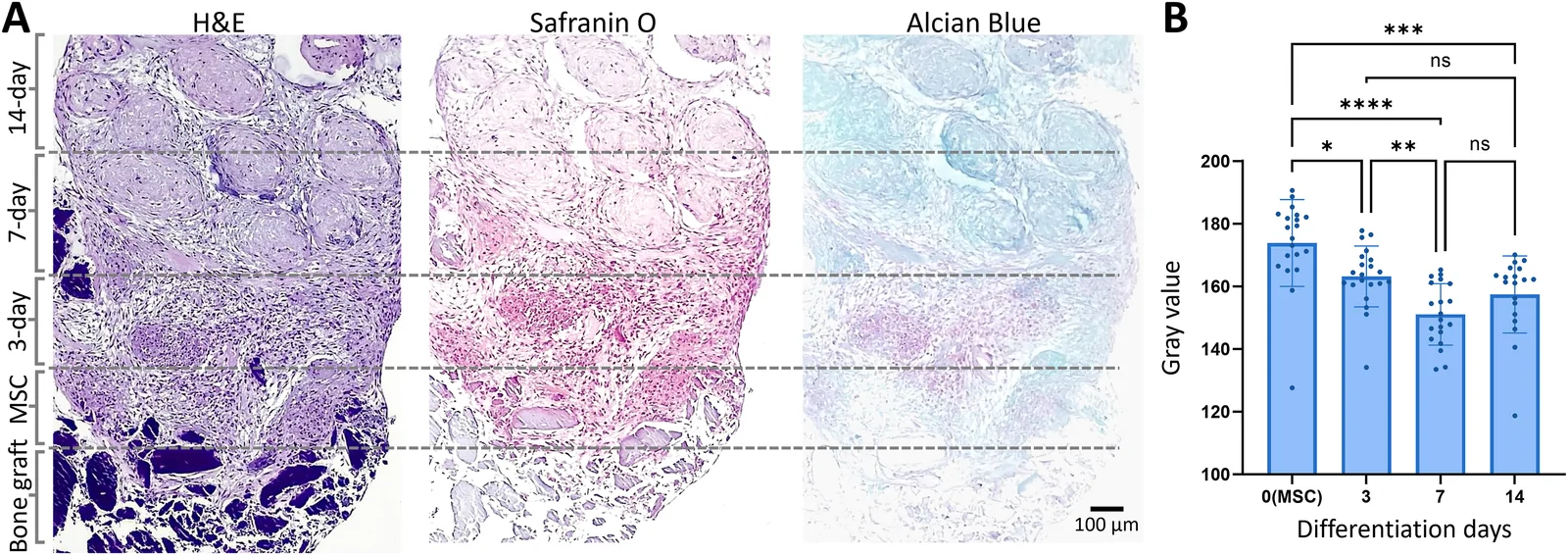
**In vitro assembly of a five-component cartilage-bone construct**. The construct was assembled, from the basal to upper regions, using a porous bone graft, undifferentiated MSCs, Day-3 chondrogenic spheroids, Day-7 chondrogenic spheroids, and Day-14 chondrogenic spheroids. Histological analyses using H&E, Safranin O, and Alcian Blue were performed following in vitro culture to evaluate structural continuity and cartilage-associated ECM deposition across the assembled construct. Regional Alcian Blue staining was quantified and is presented as mean ± SD (n = 20 measurements per region; two-tailed *t*-test; *p < 0.05, **p < 0.01, ***p < 0.001, and ****p < 0.0001).”.

Incorporation of undifferentiated MSCs at the bone interface markedly enhanced cellular infiltration into the porous bone substitute, supporting improved osteochondral integration (Fig. 5). When chondrogenic spheroids were assembled adjacent to deproteinized bovine bone, the presence of undifferentiated MSCs at the subchondral interface promoted intimate contact between the neocartilaginous tissue and the bone matrix (Fig. 5A). Histological analysis demonstrated active MSC migration into the interstitial spaces of the bone scaffold, suggesting that undifferentiated MSCs function as a biologically responsive interface layer that facilitates structural continuity between chondrogenic neotissue and subchondral bone. Importantly, these findings support the concept that differentiation-stage programming can be used as a design parameter to generate hierarchical cartilage architecture.

### 2.5 Pilot evaluation of MSC-derived chondrogenic spheroid grafts in porcine cartilage defects

**Fig. 6:**
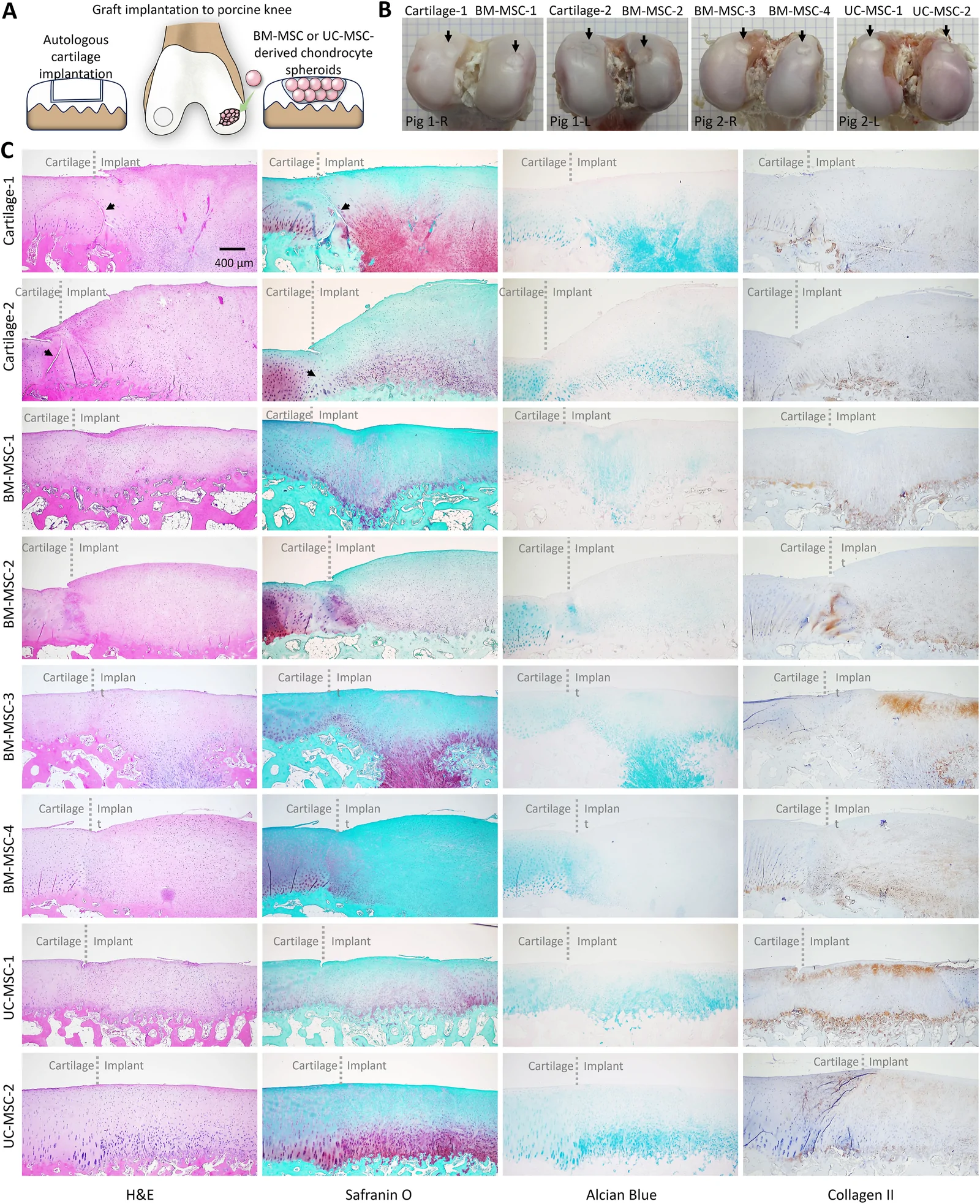
**Pilot evaluation of MSC-derived chondrogenic spheroid grafts in porcine cartilage defects**. (A) Schematic illustration of the implantation of autologous cartilage or BM-MSC- and UC-MSC-derived chondrogenic spheroid grafts into femoral-condyle defects. (B) Gross appearance of the treated femoral condyles at 6 months after implantation. Arrows indicate the treated defect sites. (C) Histological evaluation of defects treated with autologous cartilage (Cartilage-1 and Cartilage-2), BM-MSC-derived spheroid grafts (BM-MSC-1 to BM-MSC-4), or UC-MSC-derived spheroid grafts (UC-MSC-1 and UC-MSC-2). Sections were evaluated using H&E, Safranin O, and Alcian Blue staining and collagen type II immunohistochemistry. Dashed lines indicate the approximate boundaries between the adjacent native cartilage and implanted or repair tissue, and arrows indicate visible interfacial gaps. Proteoglycan staining and collagen type II immunoreactivity within the repair tissue were variable and generally less intense than those in the adjacent native cartilage, indicating incomplete matrix maturation. Scale bar, 400 μm. This exploratory pilot study included two animals in total (n = 2); individual defects and histological sections were nested within the animals and were interpreted descriptively without inferential group comparisons.

To obtain preliminary evidence regarding the feasibility of spheroid-based cartilage repair, BM-MSC-derived and UC-MSC-derived chondrogenic grafts were evaluated in a pilot porcine study involving two animals (Fig. 6A). Cylindrical cartilage defects measuring 6 mm in diameter and approximately 2 mm in depth were created in the femoral condyles. Chondrogenic MSC spheroids with defined-stage (7, 14, 21 days of chondrogenic induction) were implanted into the defect layer by layer, sequentially. The grafts didn't contain the bovine bone substitute and were therefore distinct from the five-component cartilage–bone constructs evaluated in vitro in Fig. 5.

At 6 months after implantation, repair tissue was observed within the treated defects (Fig. 6B). Safranin O and Alcian Blue staining within this tissue was generally weaker and less uniform than that of the adjacent native cartilage, indicating limited proteoglycan accumulation and incomplete matrix maturation. Collagen type II immunoreactivity was detectable but heterogeneous across the examined specimens (Fig. 6C). Some spheroid-treated interfaces appeared continuous with the adjacent cartilage, whereas visible gaps were present at some autologous cartilage implant boundaries. Because this exploratory study included only two animals, these observations are descriptive and do not establish treatment efficacy, improved integration, or superiority over autologous cartilage transplantation.

## 3 Discussion

The regeneration of articular cartilage requires not only restoration of extracellular matrix composition but also reconstruction of hierarchical organization and stable integration with host tissue [33]. In this study, we demonstrate that the maturation stage of MSC-derived chondrogenic spheroids functions as a programmable design parameter, governing extracellular matrix remodeling, spheroid fusion dynamics, and ultimately hierarchical tissue assembly. By leveraging differentiation-stage heterogeneity, we established a bottom-up strategy for constructing stage-defined hierarchical cartilage grafts with enhanced interfacial integration in a large-animal model.

Previous bottom-up studies established that mesenchymal condensations, micropellets, spheroids, and organoids can be assembled to generate stratified cartilage and spatially defined osteochondral tissues [[22], [23], [24], [25], [26], [27]]. For example, developmentally programmed organoids representing early, mature, and prehypertrophic cartilage phenotypes have been spatially arranged to produce predetermined cartilage- and bone-like regions following ectopic implantation [27]. More recent studies have shown that spheroid maturation, matrix remodeling, microscaffold incorporation, and microtissue packing density can substantially influence fusion and graft development [[28], [29], [30], [31]]. Accordingly, the novelty of the present study does not lie in the first assembly of zonally defined cartilage, but in examining differentiation stage as an intrinsic biofabrication variable. We quantified stage-dependent spheroid fusion, observed persistent inter-spheroid boundaries associated with advanced matrix maturation, and found that incorporating immature MSC populations promoted the integration of mature chondrogenic spheroids. This principle was subsequently applied to the fabrication of stage-defined multilayered cartilage constructs. The orthotopic porcine experiment provided a preliminary assessment of surgical feasibility, graft retention, safety, and tissue-interface responses. However, because this pilot study included only two animals, it was not designed to establish regenerative efficacy, statistically validate host–graft integration, or demonstrate superiority over autologous cartilage transplantation. The principal contribution of this work is therefore the identification of a functional relationship among differentiation stage, matrix maturation, remodeling capacity, and spheroid fusion, together with a developmentally informed strategy for improving interfacial continuity during hierarchical cartilage bioassembly.

Our transcriptomic analysis revealed that chondrogenic maturation is accompanied by progressive enrichment of extracellular matrix assembly and remodeling programs (Fig. 2). BM-MSC–derived spheroids displayed stronger induction of collagen fibril organization and hypertrophy-associated genes (e.g., COL10A1, MMP13), whereas UC-MSC–derived spheroids exhibited comparatively regulated matrix programs with reduced hypertrophic signatures. Importantly, these molecular differences were paralleled by functional differences in fusion kinetics. Early-stage spheroids demonstrated rapid boundary dissolution and shape relaxation, whereas mature spheroids exhibited persistent interfaces and delayed fusion (Fig. 3). Incorporation of immature MSC populations restored fusion capacity of mature spheroids. These findings indicate that differentiation stage influences not only matrix composition but also the dynamic remodeling capacity required for inter-spheroid integration. Thus, maturation state represents a programmable parameter that can be strategically deployed to balance structural stability and integration potential within engineered constructs.

The stage-dependent fusion behavior observed in this study is likely governed by multiple interacting mechanisms. Progressive chondrogenic maturation increases ECM accumulation and the stiffness and viscosity of cartilaginous spheroids, and these biomechanical changes are associated with altered fusion kinetics [31,32,43,44]. A denser and less deformable matrix may consequently constrain contact-area expansion, cellular rearrangement, and matrix interpenetration between adjacent spheroids. Matrix viscoelasticity also regulates MSC migration and spheroid fusion [22,45] through cytoskeletal pathways involving ROCK and Rac1 [45]. In parallel, differentiation-associated changes in integrin-mediated adhesion and integrin/FAK/ERK signaling may influence mechanotransduction, cytoskeletal organization, and interfacial remodeling [46]. Cadherin-mediated cell–cell adhesion, particularly N-cadherin-dependent mesenchymal condensation, matrix-degrading enzymes, and microtissue packing density may act together to regulate fusion. Less mature MSC populations may therefore facilitate integration by retaining greater migratory and remodeling capacity [31,45,47]. However, these mechanisms were not directly examined in the present study.

The hierarchical matrix distribution observed after assembly is consistent with the stage-associated phenotypes established before construct fabrication. Because Day-3, Day-7, and Day-14 spheroids were differentiated separately before being spatially arranged, each layer entered the construct with a distinct developmental history and ECM state. This interpretation is supported by previous studies in which phenotypically programmed cartilaginous organoids or microtissues retained their regional functions after assembly and generated spatially organized cartilage or osteochondral tissues in vivo [27,42]. Our approach leverages the same modular principle while incorporating differentiation-stage-dependent fusion behavior and an immature MSC layer to promote integration.

Nevertheless, spatial organization within a multicellular construct is not determined solely by the properties of its initial building blocks. Oxygen and nutrient gradients can develop within MSC spheroids and larger engineered tissues, with their magnitude depending on aggregate size, cellular density, and the culture platform [48]. The porous bone substitute may also influence transport, cell migration, and matrix anchorage at the basal interface. Therefore, although the correspondence between the preassembly phenotypes and their predefined positions supports a contribution from stage-specific programming, the current study cannot fully separate this contribution from emergent microenvironmental effects. We have consequently moderated our conclusions and describe the constructs as spatially organized assemblies of stage-defined spheroids rather than asserting that differentiation stage alone drives zonal organization.

Compared with multilayered hydrogels, porous scaffolds, and bioprinted constructs, spheroid-based assembly offers several advantages. First, spheroids smaller than 500 μm mitigate necrotic core formation and improve mass transport during in vitro culture [18,19]. Second, reliance on cellular self-organization reduces the volumetric burden of exogenous biomaterials, potentially lowering inflammatory responses [49] and foreign body reactions [50,51]. Third, differentiation-stage programming allows functional stratification without requiring distinct biomaterial compositions for each layer. Importantly, prior organoid-based osteochondral systems demonstrated zone-specific functionality [27]; however, differentiation-stage heterogeneity was not explicitly utilized as a programmable parameter to regulate fusion and integration. Our results extend bottom-up cartilage engineering by identifying maturation stage as a controllable variable that links molecular phenotype to assembly mechanics.

Native articular cartilage exhibits depth-dependent differences in cellular morphology, matrix composition, collagen organization, and mechanical function. Its superficial zone contains flattened chondrocytes, relatively low GAG content, abundant tangentially aligned collagen, and PRG4/lubricin, whereas deeper zones contain progressively more proteoglycans and distinct cellular and collagen orientations. Calcified cartilage provides continuity with subchondral bone [1,2]. The present constructs reproduce selected features of this hierarchy, including spatially defined cellular building blocks, regional differences in GAG and collagen type II deposition, cartilage-associated ECM formation, and a cellular interface with a bone substitute.

However, the observed matrix pattern does not fully reproduce native zonal cartilage. Day-14 spheroids in the upper region displayed greater GAG deposition, opposite to the relatively low GAG content of the native superficial zone. Moreover, collagen type II staining does not establish the high collagen density or tangential fiber alignment of that zone. Cellular morphology, radial-zone collagen orientation, and a mineralized calcified-cartilage interface were not evaluated. Thus, “multizonal” refers to the assembly of spheroids with distinct differentiation histories and matrix phenotypes rather than complete replication of the four anatomical zones. Future studies should refine the layer arrangement to generate a PRG4-rich, low-GAG superficial region and an aggrecan-rich deep region. Depth-resolved molecular, collagen-orientation, mechanical, and frictional analyses will be required to determine whether these constructs reproduce native zonal structure and function.

The porcine experiment was an exploratory pilot study with a limited animal-level sample size of two pigs. Although multiple defects and histological sections were examined, these observations were nested within the same animals and did not constitute independent biological replicates. Consequently, formal semiquantitative and inferential statistical comparisons were not performed, and the findings should be interpreted as preliminary feasibility observations. Accordingly, the in vivo findings support the feasibility of spheroid-derived cartilage implantation and suggest potential integration with adjacent cartilage. MSC-derived spheroid constructs exhibited improved continuity with native cartilage compared to autologous cartilage implantation, which displayed persistent boundary gaps. This enhanced integration is consistent with the in vitro fusion data, suggesting that stage-dependent remodeling capacity contributes to interfacial bonding in vivo. Future adequately powered studies should compare precisely defined cartilage-only and cartilage–bone assemblies and should document the preimplantation spatial composition, dimensions, mechanical properties, and stability of each graft.

The classification of the defects also requires caution. Although the defects were intended to be full-thickness chondral defects, their target depth of approximately 2 mm may have resulted in focal disruption of the subchondral plate. The bone-like repair tissue observed in some sections may therefore reflect subchondral bone remodeling, hypertrophic maturation and endochondral ossification of the implanted MSC-derived tissue, or both. The present histological data cannot distinguish among these possibilities. Future adequately powered studies should verify defect depth and subchondral involvement and include micro-CT, mineralization-specific staining, and markers of hypertrophic or osteogenic differentiation, such as collagen type X, RUNX2, MMP13, and osteocalcin.

Nevertheless, several limitations warrant further investigation. Quantitative mechanical testing of hierarchical constructs will be necessary to determine load-bearing capacity relative to native cartilage. Long-term evaluation beyond six months is required to assess durability and hypertrophy risk. In addition, detailed immune profiling in xenogeneic or allogeneic settings will be critical to validate the safety profile of UC-MSC–derived constructs. An untreated defect control was not included in this exploratory pilot study. Therefore, particularly given the possibility of focal subchondral plate involvement, endogenous repair cannot be distinguished from graft-associated tissue formation. Future confirmatory studies should include untreated and fibrin-glue-only defects to establish the natural healing response and determine the specific contribution of the implanted spheroid grafts.

This study identifies differentiation stage as a bioassembly parameter linking the cartilage-associated phenotype of MSC spheroids with their fusion and remodeling capacity. Early-stage spheroids fused more readily, whereas undifferentiated MSCs promoted integration of mature chondrogenic spheroids and reduced persistent boundaries. Sequential assembly generated hierarchical constructs with regional extracellular matrix differences using both BM-MSCs and UC-MSCs. The two-animal porcine pilot demonstrated implantation feasibility but showed heterogeneous proteoglycan and collagen type II staining, indicating incomplete matrix maturation. Its limited sample size precluded conclusions regarding regenerative efficacy, improved integration, or superiority over autologous cartilage implantation. These findings provide a developmentally informed framework for cartilage bioassembly. Adequately powered studies incorporating quantitative histology, mechanical testing, and controlled defect characterization are required to establish translational efficacy.

## 4 Materials and methods

### 4.1 Cell expansion and differentiation of BM-MSC and UC-MSC

Human mesenchymal stem cells from bone marrow (BM-MSC) (Cat. No. C-12974, PromoCell, Germany) and human mesenchymal stem cells from umbilical cord matrix (UC-MSC) (Cat. No. C-12971, PromoCell, Germany) were cultured in mesenchymal stem cell growth medium 2 (Cat. No. C-28009, PromoCell, Germany). Cells were incubated in 37°C humidified incubators under an atmosphere of 5% CO_2_ in air and were daily checked by morphology.

In the trilineage differentiation experiments, cells were seeded onto a 6-well plate in a growth medium overnight and then cultivated in an adipogenic or osteogenic differentiation medium, respectively. The differentiation medium was renewed every 3 days for 7 days, 14 days, and 21 days. For adipogenesis, the cells were cultured in mesenchymal stem cell adipogenic differentiation medium 2 (Cat. No. C-28016, PromoCell, Germany) and stained with Oil Red O staining kit (Cat. No. 0843, ScienCell, USA) according to manufacturer instructions. For osteogenesis, the cells were cultured in mesenchymal stem cell osteogenic differentiation medium (Cat. No. C-28013, PromoCell, Germany) and stained with Alizarin Red S staining kit (Cat. No. 0223, ScienCell, USA) according to the manufacturer's instructions.

For chondrogenesis, MSCs were cultured in 15-mL centrifuge tubes with growth medium and changed to mesenchymal stem cell chondrogenic differentiation medium (Cat. No. C-28012, PromoCell, Germany) the next day. The differentiation medium was renewed every 3 days, and the spheroids were cultured for 7 days, 14 days, and 21 days in the centrifuge tube. The chondrogenic spheroids were collected on each differentiated day and fixed with 4% PFA. The sections of the paraffin-embedded tissue blocks were stained with HE staining, Alcian Blue staining (Cat. No. 8378, ScienCell, USA), Safranin O staining (Cat. No. 8348, ScienCell, USA), and Collagen type II staining (Cat. No. ab34712, Abcam, USA) individually.

### 4.2 Flow cytometric analysis of mesenchymal stem cell surface molecules

MSCs were characterized by fluorescence-activated cell sorting (FACS) Calibur flow cytometer (BD Bioscience) using PE-anti-CD34 antibody (Cat. No. ab187284, abcam), FITC-anti-CD90 antibody (Cat. No. ab124527, abcam), and mouse IgG1 isotype control (Cat. No. ab280974, abcam). BM-MSCs or UC-MSCs (0.2 × 10^6^ cells) were incubated with PE-anti-CD34, FITC-anti-CD90, or isotype control antibodies at 4°C for 45 min. Samples were analyzed by FACS-Calibur.

### 4.3 Spheroid culture

MSCs were incubated in 1.5 mL microcentrifuge tubes at 5×10^5 cells per tube. The cells were incubated with chondrogenic differentiation medium (Cat. No. C-28012, PromoCell, Germany) at 37°C in a humidified incubator under an atmosphere of 5% CO_2_ in air. Medium was changed every 3-4 days to maintain the function of chondrogenic induction. The spheroids were collected on days 3, 7, 14, and 21 for following experiments, including histological analyses, RNAseq, fabrication of chondrogenic spheroid mosaic, spheroid fusion assay.

### 4.4 Bulk RNA sequencing and exploratory transcriptomic analysis

Bulk RNA sequencing was performed for four experimental conditions: BM-MSC-derived spheroids after 7 and 14 days of chondrogenic induction and UC-MSC-derived spheroids after 7 and 14 days of induction. For each condition, total RNA was isolated from a pooled sample of 20 spheroids to obtain sufficient RNA for sequencing. One pooled RNA sample was prepared and sequenced for each condition (n = 1 per condition). Therefore, the 20 pooled spheroids constituted one RNA-seq sample rather than independent biological replicates.

Sequencing libraries were prepared using the TruSeq Stranded mRNA Library Prep Kit (Illumina, San Diego, CA, USA) according to the manufacturer's instructions. Briefly, mRNA was purified from 1 μg of total RNA using oligo(dT)-coupled magnetic beads and fragmented at an elevated temperature. First-strand cDNA was synthesized using reverse transcriptase and random primers, followed by second-strand cDNA synthesis and adenylation of the 3′ ends. Sequencing adapters were subsequently ligated, and the libraries were purified using the AMPure XP system (Beckman Coulter, USA). Library quality was assessed using an Agilent 2100 Bioanalyzer and a real-time PCR system. Qualified libraries were sequenced on an Illumina NovaSeq 6000 platform to generate 150-bp paired-end reads. Library preparation and sequencing were performed by Genomics, BioSci & Tech Co. (New Taipei City, Taiwan).

Raw sequencing reads were processed using fastp (v0.20.0) to remove adapter sequences and low-quality bases [52]. Clean reads were aligned to the human reference genome GRCh38, Ensembl release 98, using HISAT2 (v2.1.0) [53]. Gene-level read counts were quantified using FeatureCounts (v2.0.1) [54] and normalized for descriptive comparisons. The evaluated contrasts were BM-MSC Day 14 versus Day 7, UC-MSC Day 14 versus Day 7, BM-MSC versus UC-MSC at Day 7, and BM-MSC versus UC-MSC at Day 14. Each condition in these comparisons was represented by one pooled RNA sample.

Because biological RNA-seq replicates were unavailable, within-group dispersion and biological variability could not be estimated. Formal differential-expression analysis using DESeq2 or edgeR was therefore not performed, and no adjusted p-value or false discovery rate threshold was used to define differentially expressed genes. Instead, log_2_ fold changes were calculated descriptively to rank expression differences and prioritize candidate genes and pathways. For visualization and candidate-gene prioritization, an absolute log_2_ fold change of at least 1 was used to identify genes showing notable expression changes. These genes were not considered statistically validated differentially expressed genes.

Exploratory Gene Ontology enrichment analysis and Gene Set Enrichment Analysis (GSEA) were performed using descriptive gene lists and ranked gene-expression changes. Where reported, adjusted enrichment p values represent pathway-level metrics generated by the enrichment algorithm and do not establish biologically replicated differential expression. All enrichment patterns were therefore interpreted as hypothesis-generating. Selected candidate genes were subsequently evaluated by RT-qPCR using at least four independent biological replicates per group (n ≥ 4).

### 4.5 RT-qPCR

For each experimental condition, an independent sample (a biological replicate) consisted of 20 MSC spheroids to obtain sufficient RNA (20 spheroids in one biological replicate). The Total RNA was isolated from cells by using TRIZOL Reagent (Cat. No. 15596026, ThermoFisher, USA). 2 μg of total RNA was used for transcription into 20 μL cDNA by using High-Capacity cDNA Reverse Transcription Kit (Cat. No. 4368814, ThermoFisher, USA) at the thermal cycler according to the manufacturer's instructions. Then, the cDNA mixed with Fast SYBR™ Green Master Mix (Cat. No. 4385610, ThermoFisher, USA) was amplified and detected by StepOnePlus real-time PCR System (ThermoFisher, USA). Gene expression was analyzed using the ΔΔCT method relative to undifferentiated MSCs with the reference gene GAPDH. The oligonucleotide primers for quantitative PCR were the same as described before [35]. Regarding the RT-qPCR analysis, gene expression data were obtained from at least four biological replicates, with each biological replicate analyzed in triplicate (technical replicates) during quantitative PCR.

### 4.6 Chondrogenic spheroid fusion assay

Cells were cultured in a 96-well round-bottom plate (Cat. No. 7007, Corning, USA) to form spheroids for 1, 7, and 14 days. Three Day-7 spheroids were collected and mixed with the equivalent mesenchymal stem cells for another 7 days to form a composite spheroid containing Day-7 MSCs and Day-14 spheroids. Three Day-14 spheroids were also collected and mixed with the equivalent mesenchymal stem cells for another 1 day to form a composite spheroid containing Day-1 MSCs and Day-14 spheroids. Two of the same type of spheroids were collected and cultured in a 96-well round-bottom plate.

The spheroids’ dynamic fusion was monitored by the IncuCyte S3 system (Sartorius, USA) for 24 h, using the live-cell analysis to visualize the dynamic cell-cell fusion. Morphological fusion kinetics were quantified from the normalized aspect ratio (AR) at serial time points, with five independent spheroid pairs analyzed per condition. The projection area and AR, calculated as the ratio of the minor axis to the major axis of the fusing spheroids in each frame, were quantified using ImageJ. Contact area, interface length, and fusion efficiency were not independently quantified.

Following live imaging, the paired spheroids were fixed with 4% paraformaldehyde, paraffin-embedded, sectioned, and stained with hematoxylin and eosin (H&E). Sections spanning the central contact region were examined to visualize tissue continuity and residual boundaries between the paired spheroids. Histological images were acquired under consistent magnification and imaging conditions.

Ridge-based interface visualization was performed using the Ridge Detection plugin in Fiji/ImageJ [55], as detailed in the **Supplementary Note S1**. Briefly, H&E color deconvolution was applied, and the channel providing consistent contrast between the putative interface and surrounding tissue was converted to an 8-bit image. The strongly stained outer spheroid rim was excluded, and analysis was restricted to a predefined region surrounding the expected contact zone. Ridge Detection parameters, including line width, sigma, detection thresholds, polarity, and minimum line length, were established using representative control images and then applied consistently across all groups. Detected line-like structures were superimposed on the corresponding H&E images to qualitatively visualize the persistence, fragmentation, or disappearance of the inter-spheroid boundary (Fig. S6).

### 4.7 Fabrication of 5-layer chondrogenic neotissue

For multilayer construct fabrication, MSCs were cultured in 96-well round-bottom plates (Cat. No. 7007, Corning, USA) and subjected to chondrogenic induction for 3, 7, or 14 days. Day 3 was selected as the earliest fabrication time point because the cells had formed cohesive spheroids that could be transferred and spatially positioned reproducibly. Day-7 spheroids represented an intermediate stage of chondrogenic differentiation, whereas Day-14 spheroids represented a relatively mature, matrix-rich stage with reduced fusion capacity. Day-1 spheroids were included in the fusion assay to characterize early fusion behavior but were not used for multilayer fabrication because they lacked sufficient structural stability for reproducible handling and assembly.

At each differentiation stage, 60 spheroids were collected and suspended in 5 μL of a Porcogen mixture comprising DMEM/F12 medium (Cat. No. 12400024, Gibco, USA), Porcogen atelocollagen (Cat. No. 53120208, Sunmax Biotechnology, Taiwan), and neutralizing solution at a volume ratio of 1:8:1. A 2% agarose mold containing a U-shaped cylindrical cavity with a diameter of 2 mm was used to support sequential assembly. Crushed osteoconductive bovine bone substitute (Cat. No. BAL05, Osstem Implant, China) was placed at the bottom of the mold, followed sequentially by a layer of undifferentiated MSCs, 60 Day-3 spheroids, 60 Day-7 spheroids, and 60 Day-14 spheroids at the uppermost region. The assembled constructs were maintained in MSC chondrogenic differentiation medium for an additional 7 days and then fixed with 4% paraformaldehyde. After paraffin embedding and sectioning, the constructs were evaluated using hematoxylin and eosin, Alcian Blue, and Safranin O staining, together with collagen type II immunohistochemistry.

### 4.8 Fabrication of collagen-embedded chondrogenic MSCs for porcine study

Approximately 10 mL of heparinized bone marrow was aspirated from the dorsal iliac wing (posterior iliac crest) of each Lanyu miniature pig and immediately transported to the laboratory for processing. Bone marrow-derived mesenchymal stem cells (BM-MSCs) were isolated by density-gradient centrifugation using Ficoll (Cat. No. 17-5446-52, GE Healthcare, Little Chalfont, UK). The recovered mononuclear-cell fraction was cultured in low-glucose Dulbecco's modified Eagle's medium (DMEM-LG; Cat. No. 31600, Gibco, Carlsbad, CA, USA) supplemented with 10% fetal bovine serum. Cells were maintained at 37°C in a humidified atmosphere containing 5% CO_2_. The culture medium was replaced twice weekly. Adherent cells were trypsinized and subcultured at a 1:3 split ratio once per week until sufficient cell numbers were obtained for chondrogenic differentiation and graft preparation, as previously described [35].

Porcine umbilical cord-derived MSCs (UC-MSCs) were isolated from the Wharton's jelly of umbilical cords collected aseptically from neonatal Lanyu donor pigs immediately after delivery. The cords were transported to the laboratory in sterile phosphate-buffered saline (PBS) containing 1% antibiotic-antimycotic solution. After repeated washing, each cord was opened longitudinally, and the umbilical arteries and vein were removed. The remaining Wharton's jelly and cord-matrix tissue was cut into approximately 2 × 2 mm fragments and placed in tissue-culture plates for explant outgrowth. The explants were cultured in low-glucose Dulbecco's modified Eagle's medium supplemented with 10% fetal bovine serum and 1% penicillin-streptomycin at 37°C in a humidified atmosphere containing 5% CO_2_. Cellular outgrowth was monitored microscopically. The medium was first replaced on Day 5 and subsequently every 3 days. After 7–10 days, when the adherent spindle-shaped cells reached approximately 80% confluence, the tissue fragments were removed, and the cells were detached using Accutase (Cat. No. PB180201, Procell system). Cells were reseeded at approximately 3 × 10^5^ cells per T25 flask and expanded under the same culture conditions. Early-passage UC-MSCs, preferably passages 4–6, were used for chondrogenic differentiation and graft preparation. Because the UC-MSCs were obtained from neonatal donor pigs rather than the recipient animals, they were considered allogeneic to the recipient pigs.

To prepare chondrogenic spheroids, both BM-MSCs and UC-MSCs were harvested and embedded in the Porcogen™ atelocollagen solution (3% type-I/type-III collagen, Sunmax Biotechnology, Taiwan) containing serum-free DMEM-LG only. The cells were resuspended in 0.5 mL neutralized atelocollagen solution with a final cell density of 2.6 × 10^6^ cells/mL, seeded in 24-well plates, and briefly incubated at 37°C for the gelatinization of atelocollagen to take place. Subsequently, 2 mL/well of the following chondrogenic medium was added: serum-free medium (DMEM-LG, Cat. No. 31600, Gibco) with ITS + Premix (Cat. No. 354352, Corning), 50 μg/mL L-ascorbic acid-2-phosphate (Cat. No. 49752, Sigma-Aldrich), 10^−7^ M dexamethasone (Cat. No. D4902, Sigma-Aldrich), and freshly added 10 ng/mL transforming growth factor beta-1 (TGF-β1, Cat. No. 100-21, PeproTech, Rocky Hill, N.J.). The cells were cultured in the chondrogenic medium for 7–21 days with medium changes every 3 days. Chondrogenic induction led to MSC condensation in the collagen gel, forming spheroids of collagen-embedded chondrogenic MSCs.

### 4.9 Pilot porcine cartilage-defect study

This exploratory pilot study included two Lanyu miniature pigs (n = 2 animals in total). The animal-use protocol was reviewed and approved by the Institutional Animal Care and Use Committee convened by PigModel Animal Technology Co. (IACUC approval no. PIG-112001).

The animals were fasted for at least 12 h before surgery, with unrestricted access to water. After washing and drying, sedation was induced by intramuscular administration of azaperone (3–5 mg/kg) and atropine (0.03–0.05 mg/kg), and respiratory rate was closely monitored. After 20–30 min, Zoletil 50 (3–5 mg/kg) was administered intramuscularly. After an additional 5–10 min, the animals were transferred to the operating table, positioned in the prone position, and intubated. General anesthesia was maintained using oxygen and nitrous oxide at a 2:1 ratio and a total flow rate of 2–3 L/min, together with 0.5–2% isoflurane. Anesthetic depth was continuously monitored, and the oxygen and nitrous oxide flow rates and isoflurane concentration were adjusted according to the respiratory rate, anesthetic depth, and surgical requirements. Body temperature was maintained throughout the procedure, and heart rate, respiratory rate, and body temperature were continuously monitored. Cefazolin (15 mg/kg) and meloxicam (0.4 mg/kg) were administered intramuscularly before surgery for antibiotic prophylaxis and analgesia, respectively.

After anesthesia was established, bilateral arthrotomy of the stifle joints was performed through incisions approximately 10 cm in length. Cylindrical defects measuring 6 mm in diameter and approximately 2 mm in depth were created manually in the weight-bearing regions of the medial and lateral femoral condyles using a metal biopsy punch. The defects were intended to remove the full thickness of the articular cartilage. However, because a fixed target depth of approximately 2 mm was used, focal disruption or exposure of the subchondral plate could not be excluded. The lesions were therefore classified as full-thickness cartilage defects with possible focal subchondral bone involvement.

Depending on the assigned treatment, the defects received: (1) UC-MSC-derived chondrogenic grafts, (2) BM-MSC-derived chondrogenic grafts, or (3) autologous cartilage transplantation. Autologous cartilage was harvested during the surgical procedure for transplantation into the assigned defects. The MSC spheroids with different levels of chondrogenic differentiation were implanted into the defect site layer by layer. No crushed bovine bone substitute was used in the preparation or implantation of the constructs. The Fibrin glue was applied to cover and secure the implanted tissue in all treatment groups. After implantation, the muscle and deeper tissue layers were closed using absorbable sutures, and the skin was closed using nonabsorbable sutures.

Postoperative health and wound conditions were assessed daily. Pain was evaluated daily during the first 7 days after surgery. Ketoprofen (1–3 mg/kg) and cefazolin (15 mg/kg) were administered as needed according to the animals’ postoperative conditions and clinical assessments. At 6 months after implantation, the animals were euthanized according to the approved protocol, and tissue specimens containing the treated femoral condyles were collected and fixed in formalin. The specimens were subsequently decalcified, dehydrated, embedded in paraffin, sectioned, and stained for histological evaluation.

Because only two animals were included, the histological findings were evaluated descriptively. Individual defects, tissue sections, and microscopic fields obtained from the same animal were considered nested observations and were not treated as independent biological replicates for inferential statistical analysis.

### 4.10 Histological analyses

The 5-μm-thick sections of paraffin-embedded tissue blocks from chondrocyte spheroids and 5-layer chondrogenic neotissue were deparaffinized with Xylene and rehydrated in distilled water through graded ethanol. The slides were stained with Alcian Blue staining kit (Cat. No. 8378, ScienCell, USA) and Safranin O staining kit (Cat. No. 8348, ScienCell, USA) to assess the amount of GAG accumulation in the implanted tissues. Hematoxylin and eosin staining were also performed to assess the general structure under an optical microscope. For Collagen type II staining, the antigen retrieval was carried out by heating the slides in low pH IHC antigen retrieval solution (Cat. No. 00-4955-58, eBioscience, USA) using the pressure cooker for 10 min. Endogenous peroxidase activity was quenched in a hydrogen peroxide block (Cat. No. ab236466, abcam, USA). The slides were then incubated with anti-Collagen type II antibody (Cat. No. ab34712, abcam, USA) at 1:200 dilution and anti-Collagen type II antibody (Cat. No. ab34712, abcam, USA for MSC spheroids) at 1:200 dilution and anti-Collagen type II antibody (rabbit polyclonal, 1:200, Cat. No. 28459-1-AP, Proteintech, USA for porcine slices) at 1:200 dilution at 4°C overnight and further incubated with goat anti-rabbit HRP-conjugate (Cat. No. ab236466, abcam, USA) for 30 min at room temperature. Finally, antigen-antibody complexes were detected by DAB (Cat. No. ab236466, abcam, USA). The staining results of the samples were observed under an optical microscope (Olympus CX43), and the pictures were taken by the built-in camera (Model: BC1200, YuanLi).

### 4.11 Spheroid staining result - color intensity analysis

To further quantify the color intensity of the staining results, Fiji/ImageJ (an open-source image processing package based on ImageJ2) was used for the analysis. The staining results of the spheroids were observed under an optical microscope, and the pictures were taken by the built-in camera. The images were analyzed using Fiji/ImageJ. Briefly, the images of Alcian Blue, Safranin O staining, and IHC staining were processed with the built-in function-color deconvolution to split the channels of the staining results into three determined colors [56].

For the Alcian Blue staining results, the vector- Alcian Blue & Hematoxylin was chosen. Alcian Blue staining was quantified using Fiji/ImageJ. Following color deconvolution, the relevant staining channel was converted into an 8-bit image, with pixel values ranging from 0 (black) to 255 (white). ROIs measuring 115 × 115 pixels were selected for analysis. Because stronger staining produces a darker image, a lower mean gray value indicates greater Alcian Blue staining intensity and greater GAG-associated matrix deposition. Thirty ROIs were evaluated within each of three independent spheroids per group, yielding 90 ROI-level measurements per group. The ROI measurements were considered subsampling observations nested within spheroids rather than independent biological replicates. Statistical analysis was performed using a mixed-effects model in which the experimental group was included as a fixed effect and spheroid identity as a random effect. This model accounted for the intra-spheroid correlation among ROI measurements while retaining the spatial information provided by all 90 ROIs. The biological sample size was n = 3 independent spheroids per group.

The vector- Masson Trichrome was used for Safranin O staining results, while the vector- Hematoxylin, DAB was selected for IHC staining results, such as Collagen II IHC staining. Random ROIs (8-bit image with the size of 115*115 pixels) were chosen for the intensity value analysis. A greater mean value leads to lower color intensity, which means a brighter color.

### 4.12 Statistics

Sample sizes are reported separately for each experiment in the corresponding figure legends. The independent experimental unit was defined according to the experimental design: an independent biological RNA sample for RT-qPCR, RNA sequencing, a spheroid pair for fusion analysis, an independently generated spheroid or construct for histological analysis, and an individual animal for the porcine study. Multiple ROIs, microscopic fields, or histological sections obtained from the same biological sample were considered subsampling measurements and were not treated as independent biological replicates. The porcine experiment was an exploratory pilot study involving two animals (n = 2), and its histological findings were interpreted descriptively.

The statistical analyses were performed using one-way analysis of variance (ANOVA). In one-way ANOVA, multiple comparisons were performed based on the Tukey method. A two-tailed *t*-test was used to test the difference. The statistical significance of the t-tests was set as p-value < 0.05 (with *, p < 0.05; **, p < 0.01; ***, p < 0.001; ****, p < 0.0001). All statistical analyses were performed either using Microsoft Excel or Prism.

Differences in Alcian Blue staining gray values among spheroids cultured for 7, 14, and 21 days under chondrogenic induction were evaluated using a nested one-way ANOVA. Chondrogenic induction duration was specified as the fixed effect, while spheroid identity was nested within induction duration to account for correlations among ROIs obtained from the same spheroid. Thirty ROIs were analyzed within each of three independent spheroids per duration, yielding 90 ROI-level measurements per group. ROI measurements were treated as within-spheroid subsamples and not as independent biological replicates; therefore, the biological sample size was n = 3 spheroids per duration. Following the omnibus nested one-way ANOVA, Tukey's multiple-comparisons test was used for pairwise comparisons between Days 7, 14, and 21. Adjusted p values are reported, with p < 0.05 considered statistically significant.

## CRediT authorship contribution statement

**Yen-Liang Liu:** Writing – review & editing, Supervision, Funding acquisition, Conceptualization, Writing – original draft. **Yun-Chieh Lin:** Writing – original draft, Methodology, Investigation, Data curation. **Yi-Chen Tsai:** Visualization, Investigation, Data curation. **Chun-Che Yen:** Investigation. **Chun-Yi Chiang:** Investigation, Data curation. **Pen-Jung Hung:** Methodology, Investigation. **Yu-Ting Huang:** Investigation, Data curation. **Chih-Hung Chang:** Validation, Investigation. **Feng-Huei Lin:** Validation, Resources. **Hwa-Chang Liu:** Validation, Supervision, Conceptualization, Writing – review & editing.

## Declaration of competing interest

The authors declare the following financial interests/personal relationships which may be considered as potential competing interests: Yen-Liang Liu reports financial support was provided by National Science and Technology Council. Yen-Liang Liu reports financial support was provided by Ministry of Science and Technology. Chun-Che Yen reports a relationship with Kartigen Biomedical Inc. that includes: employment. If there are other authors, they declare that they have no known competing financial interests or personal relationships that could have appeared to influence the work reported in this paper.

## Data Availability

Data will be made available on request.
