# Supplementary material for: Stage-specific MSC spheroid fusion enables bottom-up formation of stage-defined hierarchical cartilage graft
Source: Mater Today Bio. 2026 Sep 4;40:103649. doi: 10.1016/j.mtbio.2026.103649 (PMC13579038; doi:10.1016/j.mtbio.2026.103649)
Supplement: Supplementary file 1 [file mmc1.pdf]

Supplementary Information

Stage-specific MSC spheroid fusion enables bottom-up formation of stage-defined hierarchical cartilage graft

Yen-Liang Liu[1,2,3,*], Yun-Chieh Lin[4], Yi-Chen Tsai[5], Chun-Che Yen[6], Chun-Yi Chiang[7], Pen-Jung Hung[2], Yu-Ting Huang[1], Chih-Hung Chang[8,9], Feng-Huei Lin[7,10,11], Hwa-Chang Liu[4,12,*]

[1]Master Program for Biomedical Engineering, College of Biomedical Engineering, China Medical University, Taichung City 406040, Taiwan
[2]Cancer Biology and Precision Therapeutics Center, China Medical University, Taichung City 406040, Taiwan
[3]Graduate Institute of Biomedical Sciences, College of Medicine, China Medical University, Taichung City 406040, Taiwan
[4]Department of Orthopaedic Surgery, National Taiwan University Hospital, Taipei City 100225, Taiwan
[5]Department of Biomedical Engineering, College of Biomedical Engineering, China Medical University, Taichung City 406040, Taiwan
[6]Kartigen Biomedical Inc., Taipei City 100047, Taiwan
[7]Ph.D. Program for Aging, College of Medicine, China Medical University, Taichung City 406040, Taiwan
[8]Department of Orthopaedic Surgery, Far Eastern Memorial Hospital, New Taipei City 220216, Taiwan
[9]Graduate School of Biotechnology and Bioengineering, Yuan Ze University, Taoyuan City 320315, Taiwan
[10]Department of Biomedical Engineering, College of Engineering, National Taiwan University, Taipei City 106319, Taiwan
[11]Institute of Biomedical Engineering and Nanomedicine, National Health Research Institutes, Miaoli County 350401, Taiwan
[12]Department of Orthopaedic Surgery, Taiwan Adventist Hospital, Taipei City 105404, Taiwan
*Correspondence: hcliu8@gmail.com, allen.liu@cmu.edu.tw

Content

Fig. S1 | Cell surface biomarkers and cell viability of chondrogenic BM-MSC and UC-MSC. 2
Fig. S2 | Transcriptomic profiling of chondrogenic MSCs. 3
Fig. S3 | BM-MSC-derived chondrogenic spheroid mosaic. 4
Fig. S4 | UC-MSC-derived chondrogenic spheroid mosaic. 5
Fig. S5 | Image quantification of chondrogenic spheroid mosaic with Alcian Blue staining. 6
Note S1 | Ridge-based visualization of the histological interface between paired spheroids 7
Fig. S6 | Ridge-based visualization of the histological interface within fused spheroids 11

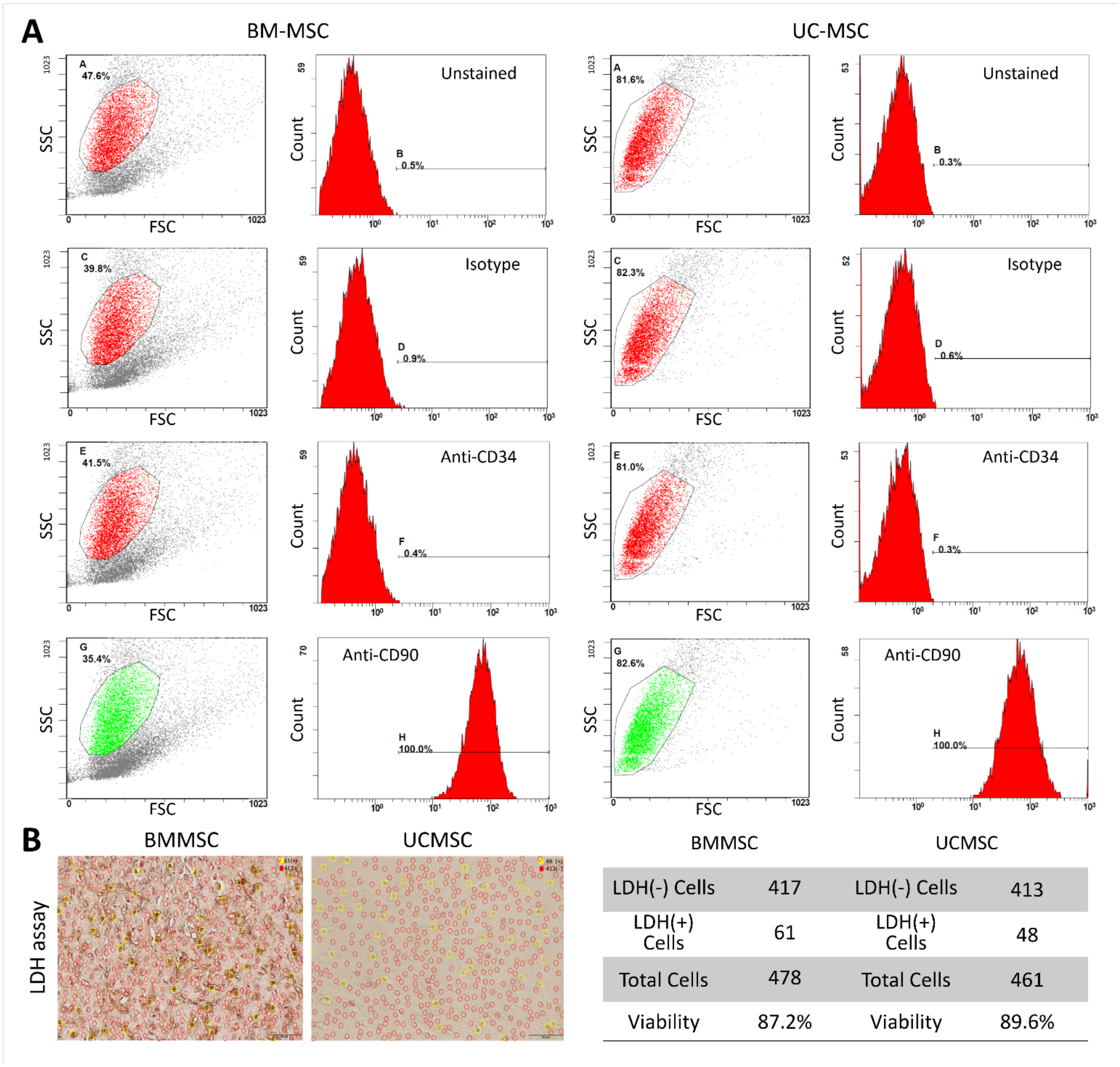

	BMMSC		UCMSC
LDH(-) Cells	417	LDH(-) Cells	413
LDH(+) Cells	61	LDH(+) Cells	48
Total Cells	478	Total Cells	461
Viability	87.2%	Viability	89.6%

Fig. S1 | Cell surface biomarkers and cell viability of chondrogenic BM-MSC and UC-MSC.
(A) Flow cytometric analysis of MSC biomarkers of BM-MSC and UC-MSC. (B) Cell viability of chondrogenic MSCs in collagen was evaluated using the LDH assay.

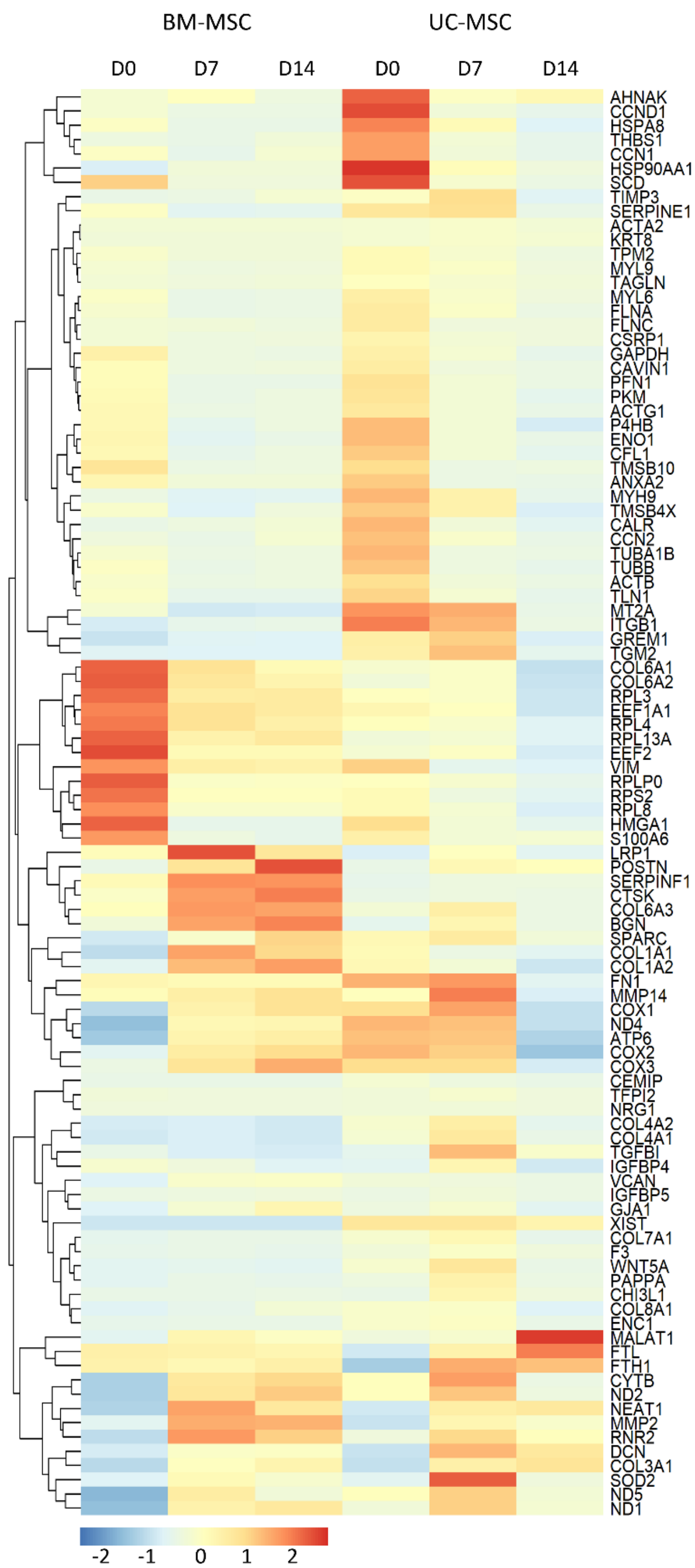

Fig. S2 | Transcriptomic profiling of chondrogenic MSCs.

mRNA sequencing was performed to investigate the transcriptomic profiles of MSCs and chondrogenic MSCs for 7 and 14 days of chondrogenic induction.

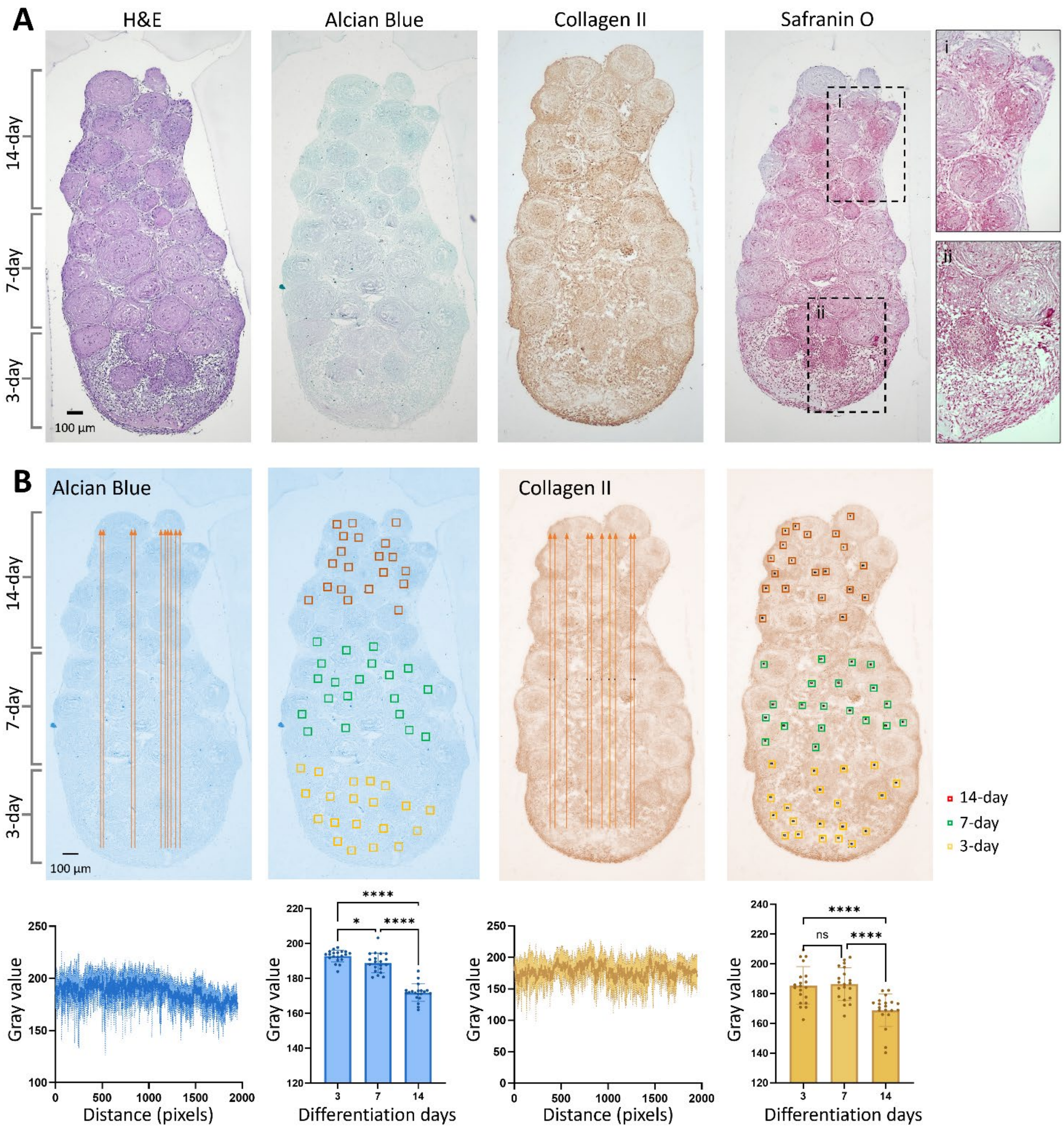

Fig. S3 | BM-MSC-derived chondrogenic spheroid mosaic.

(A) Histological analyses of spheroid stacks derived from BM-MSC using specific staining: Alcian Blue, anti-Collagen II immunohistochemistry, and Safranin O. (B) Image quantification of Alcian blue staining and collagen type II immunostaining. Longitudinal and zonal image analyses were performed to quantify the expression of glycosaminoglycans and collagen type II in BM-MSC-derived chondrogenic grafts. The gray values were measured by Image J and presented as means ± SD (n = 20 measurements/slide for zonal image analyses. Two-tailed t-test; *, $p < 0.05$; **, $p < 0.01$; ***, $p < 0.001$; ****, $p < 0.0001$). Lower gray values indicate darker blue or brown colors (greater protein expression).

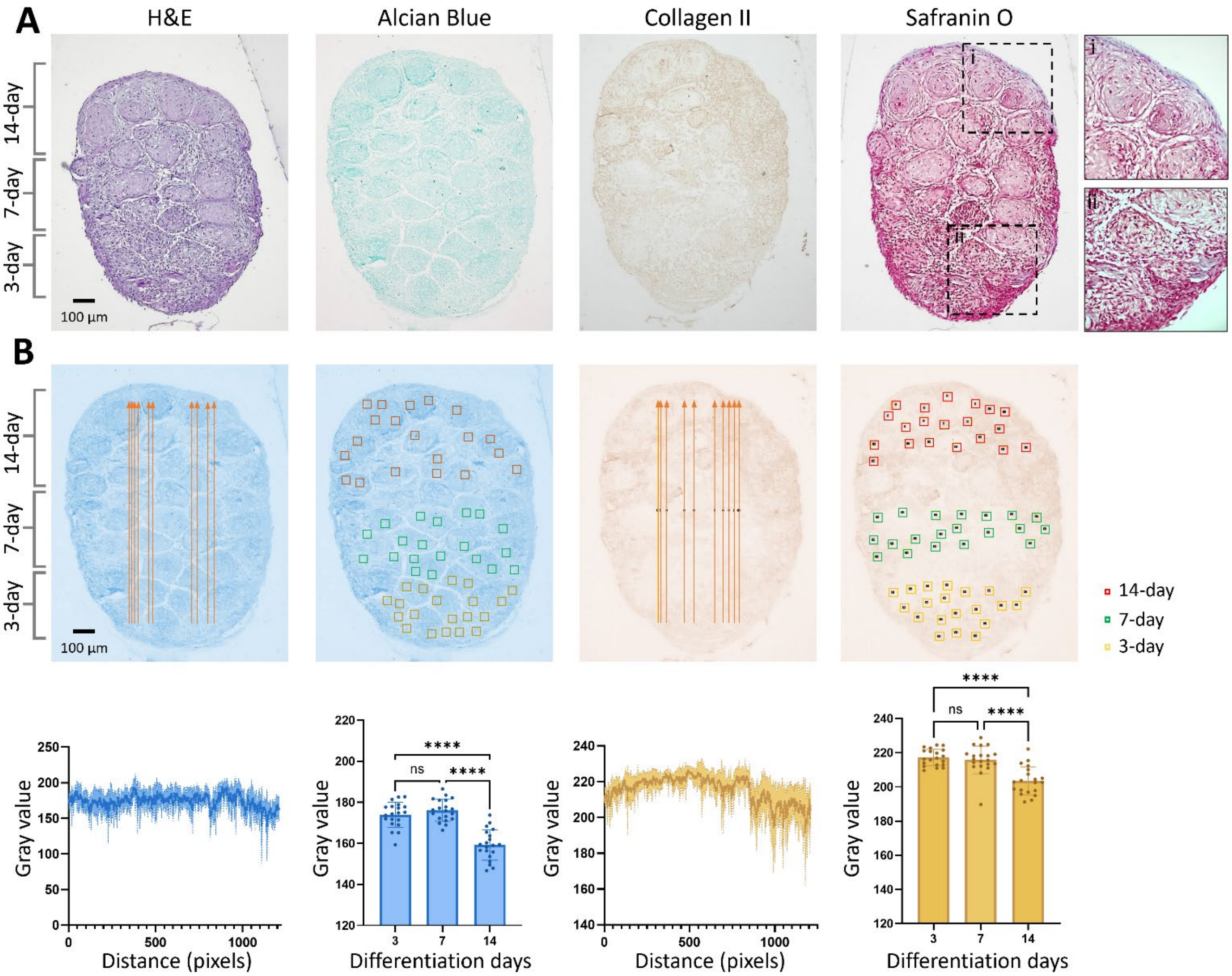

Fig. S4 | UC-MSC-derived chondrogenic spheroid mosaic.

(A) Histological analyses of spheroid stacks derived from UC-MSC using specific staining: Alcian Blue, anti-Collagen II immunohistochemistry, and Safranin O. (B) Image quantification of Alcian blue staining and collagen type II immunostaining. Longitudinal and zonal image analyses were performed to quantify the expression of glycosaminoglycans and collagen type II in UC-MSC-derived chondrogenic grafts. The gray values were measured by Image J and presented as means ± SD (n = 20 measurements/slide for zonal image analyses. Two-tailed t-test with *, $p < 0.05$; **, $p < 0.01$; ***, $p < 0.001$; ****, $p < 0.0001$). Lower gray values indicate darker blue or brown colors (greater protein expression).

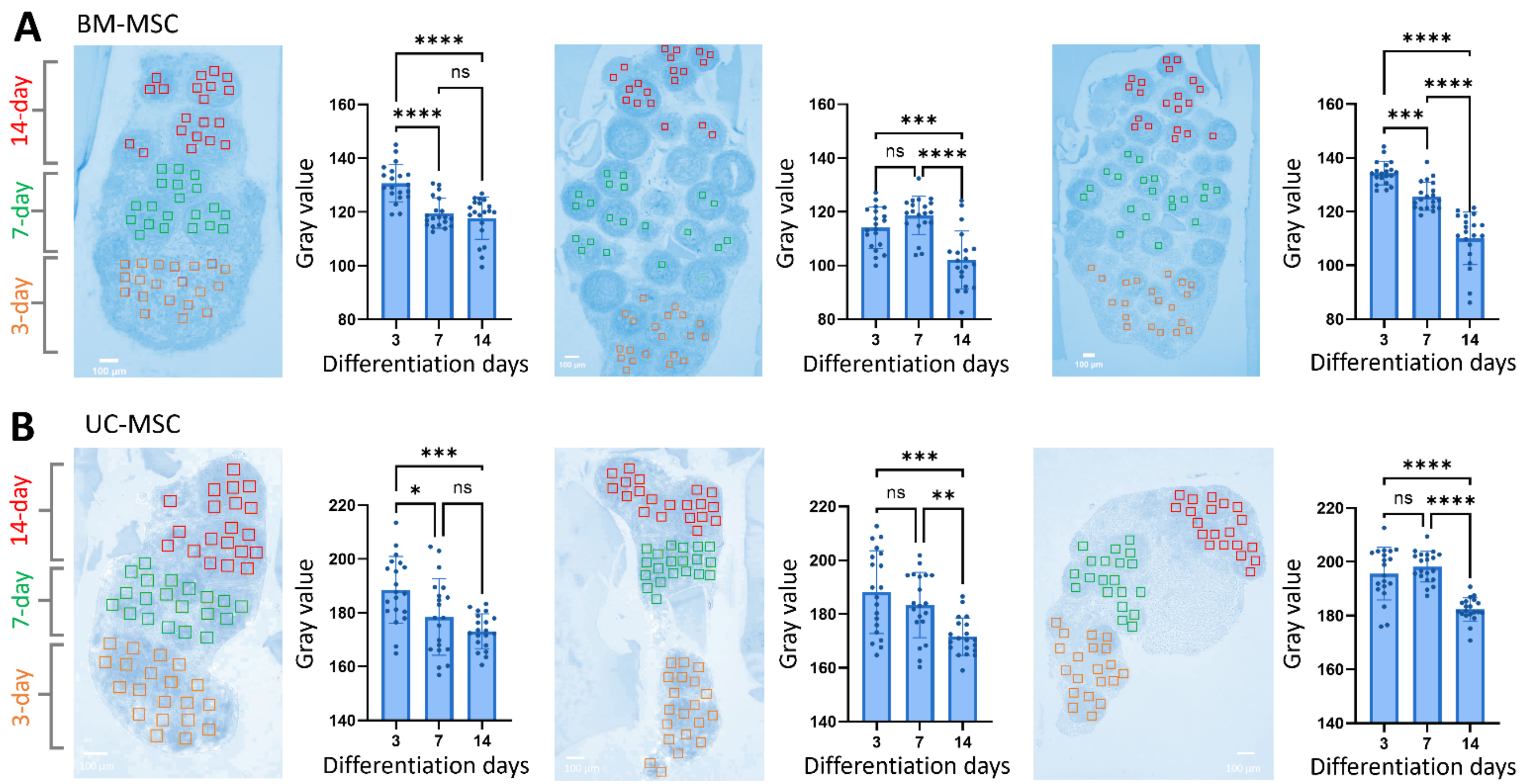

Fig. S5 | Image quantification of chondrogenic spheroid mosaic with Alcian Blue staining.
(A) Image quantification of Alcian blue staining of more BM-MSC- and UC-MSC-derived spheroid mosaic. Zonal image analyses were performed to quantify the expression of glycosaminoglycans. The gray values were measured by Image J and presented as means ± SD (n = 20 measurements/slide; two-tailed t-test with *, $p < 0.05$; **, $p < 0.01$; ***, $p < 0.001$; ****, $p < 0.0001$).

Note S1 | Ridge-based visualization of the histological interface between paired spheroids

Purpose and analytical rationale

Ridge Detection was applied to H&E-stained sections as an image-processing aid for visualizing residual line-like boundaries between paired spheroids. The analysis was intended to support qualitative assessment of interface persistence and tissue bridging. It was not used to calculate residual-interface occupancy, a histological fusion index, or other quantitative fusion parameters.

Histological preparation and image acquisition

Following the paired-spheroid fusion assay, constructs were fixed with 4% paraformaldehyde, paraffin-embedded, sectioned at 5 μm, and stained with H&E. Serial sections spanning the central contact region were examined because a single two-dimensional section may not represent the entire three-dimensional interface. Images were acquired at identical magnification, illumination, white-balance, and exposure settings using an optical microscope (Olympus CX43), and the pictures were taken by the built-in camera (Model: BC1200, YuanLi). Images were coded before processing to minimize observer bias.

Image preprocessing

Images were processed using Fiji/ImageJ. Spatial calibration was performed using the microscope calibration information. H&E color deconvolution was applied using the built-in H&E vector, and the channel providing the most consistent contrast between the putative interface and surrounding tissue was selected before group comparison. The same channel was subsequently used for all images. The selected channel was converted to an 8-bit grayscale image, and identical background-correction settings were applied across the dataset.

The spheroid outline was identified from the original H&E image. The strongly stained outer rim was excluded because it could produce a prominent ridge unrelated to inter-spheroid fusion. A region of interest was then manually defined around the expected contact zone based on the paired-spheroid geometry and the corresponding unprocessed H&E image. Ridge analysis and interpretation were restricted to this predefined interface region.

Ridge Detection

Line-like structures were identified using the Ridge Detection plugin (version1.4.0) for Fiji/ImageJ, which implements Steger's curvilinear-structure detection algorithm [1]. Detection polarity was configured to identify the residual interface as a dark line. The expected line width, sigma, lower threshold, upper threshold, and minimum line length were initially optimized using representative control images. The final settings were fixed before analysis and applied without modification to all experimental groups:

- Expected line width: 3.5 pixels
- Sigma: 1.51
- Lower threshold: 3.06
- Upper threshold: 7.99
- Detection polarity: dark line

- Minimum line length: 0 pixels

Detected ridges were added to the ImageJ ROI Manager and superimposed on the corresponding H&E images. The overlays were visually inspected alongside the original images to distinguish the putative inter-spheroid boundary from internal cellular or extracellular-matrix structures. Only ridge traces located within the predefined interface region were considered.

Qualitative interpretation

The ridge overlays were interpreted qualitatively according to boundary continuity and tissue bridging. A prominent and continuous ridge along the contact zone was considered consistent with persistence of the original inter-spheroid boundary and limited fusion. A fragmented or discontinuous ridge accompanied by tissue bridges was considered indicative of partial boundary remodeling. Minimal or absent ridge detection across multiple serial sections was considered consistent with extensive interface remodeling. Complete biological fusion was not concluded solely from the absence of a detected ridge because detection can be influenced by sectioning plane, staining contrast, tissue orientation, and image-processing parameters.

Ridge Detection was therefore used as a visualization tool complementary to live-imaging measurements of spheroid aspect ratio. The analysis identifies the persistence of line-like histological boundaries but does not directly measure mechanical integration, cellular intermixing, matrix continuity, or three-dimensional fusion.

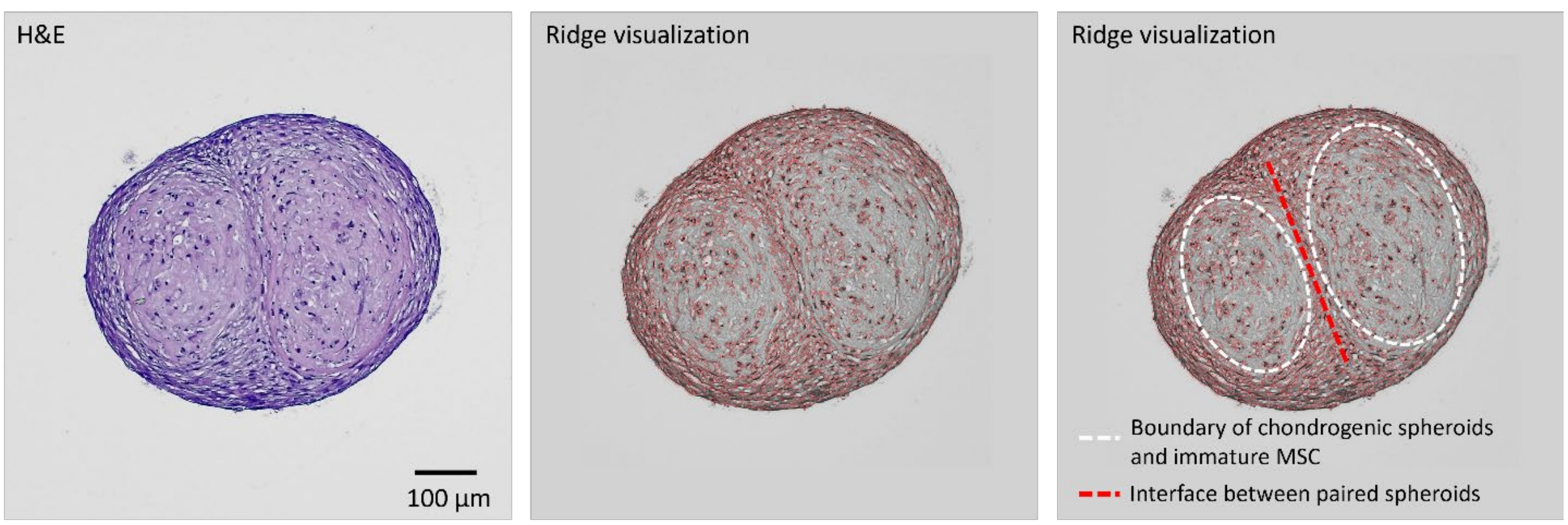

Representative H&E-stained section of paired chondrogenic spheroids surrounded by an immature MSC-derived cellular layer (left). Ridge Detection was applied to visualize line-like histological structures, which are shown as red overlays (middle). The annotated image (right) identifies the approximate boundaries between the two chondrogenic spheroid cores and the surrounding immature MSC-derived tissue (white dashed lines), as well as the residual interface between the paired spheroids (red dashed line). The continuous outer cellular envelope and tissue bridging around the spheroids indicate incorporation of immature MSCs, whereas the remaining central interface suggests that fusion was incomplete at the examined time point. Ridge Detection was used for qualitative visualization and not as a quantitative measurement of fusion. Scale bar = 100 µm.

Supplementary reference

1. Steger, C., An unbiased detector of curvilinear structures. IEEE Transactions on Pattern Analysis and Machine Intelligence, 1998. 20(2): p. 113–125.

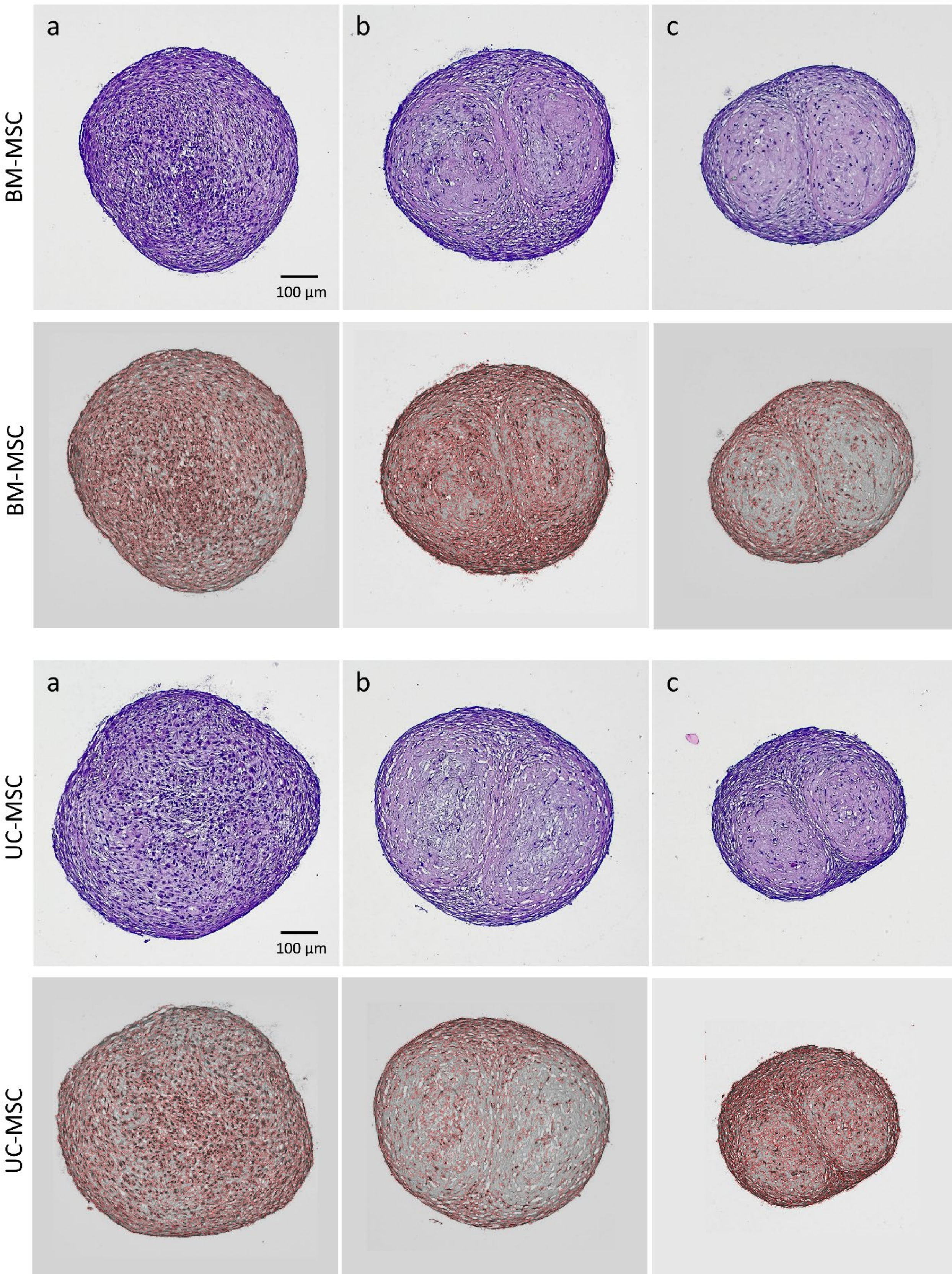
a
b
c
BM-MSC
100 µm
BM-MSC
a
b
c
UC-MSC
100 µm
UC-MSC

Fig. S6 | Ridge-based visualization of the histological interface within fused spheroids

Representative H&E-stained sections and corresponding Ridge Detection overlays of paired spheroids derived from BM-MSCs (upper panels) and UC-MSCs (lower panels) after 24 h of fusion culture. Paired spheroids were analyzed after (a) 1, (b) 7, or (c) 14 days of chondrogenic induction. Red overlays indicate line-like histological structures identified using the Ridge Detection plugin. Early-stage spheroids exhibited a more homogeneous tissue architecture with minimal residual separation, whereas Day-7 and particularly Day-14 spheroids retained more discernible central interfaces between the paired units. Ridge Detection was used for qualitative visualization of interface persistence and not for quantitative calculation of fusion. Scale bars, 100 μm.
